# Auranofin Potentiates Cisplatin Response in the Setting of Context-Dependent NOTCH-Associated Signaling States in Endometrial Cancer

**DOI:** 10.1158/2767-9764.CRC-26-0404

**Published:** 2026-10-16

**Authors:** Robert J. Lake, Cynthia Tshibangu, Niko J. Candia, Quinn U. Abfalterer, Irina V. Lagutina, Christine Pauken, Kimberly K. Leslie, Mara P. Steinkamp, Hua-Ying Fan

**Affiliations:** 1 Program in Cell and Molecular Oncology, University of New Mexico Comprehensive Cancer Center, University of New Mexico Health Sciences Center, Albuquerque, New Mexico.; 2 Division of Molecular Medicine, Department of Internal Medicine, University of New Mexico Health Sciences Center, Albuquerque, New Mexico.; 3 Biomedical Sciences Graduate Program, University of New Mexico Health Sciences Center, Albuquerque, New Mexico.; 4 Animal Models Shared Resource, Comprehensive Cancer Center, University of New Mexico, Albuquerque, New Mexico.; 5 Department of Pathology, University of New Mexico School of Medicine, Albuquerque, New Mexico.

## Abstract

Therapeutic resistance remains a major challenge in advanced and recurrent endometrial cancer. Aberrant NOTCH signaling has been associated with aggressive tumor behavior and therapeutic resistance across multiple malignancies, yet its therapeutic significance in endometrial cancer remains incompletely defined. We investigated whether auranofin (AuR), a pleiotropic gold-containing compound that inhibits thioredoxin reductase and can disrupt RBPJ chromatin occupancy, alters platinum responsiveness in endometrial cancer models. Upper-quartile *NOTCH3* copy number was associated with poorer overall survival in The Cancer Genome Atlas-Uterine Corpus Endometrial Carcinoma cohort, although this association was not retained with median-based stratification, continuous Cox proportional hazards modeling, or multivariable adjustment for clinicopathologic and molecular context. AuR treatment reduced RBPJ occupancy at canonical NOTCH target loci, including *HES1* and *HES4*, across multiple endometrial cancer models, although this was not accompanied by significant changes in steady-state *HES1* or *HES4* transcript levels in AN3CA or ARK-1 cells. Stable NOTCH3 depletion produced context-dependent effects on AuR responsiveness and significantly enhanced cisplatin (CDDP) sensitivity in AN3CA cells. Pharmacologic AuR treatment similarly potentiated CDDP response in AN3CA cells and xenografts, resulting in reduced tumor burden and prolonged endpoint-free survival following combination treatment. In contrast, ARK-1 xenografts demonstrated limited additional benefit from combined AuR plus CDDP therapy despite detectable suppression of RBPJ occupancy. Together, these findings identify context-dependent NOTCH-associated therapeutic vulnerabilities in endometrial cancer and support further investigation of AuR-based platinum-sensitization strategies in biologically defined tumor contexts.

**Significance:**

AuR disrupts RBPJ chromatin occupancy and enhances CDDP response in biologically distinct endometrial cancer contexts.

## Introduction

Endometrial cancer is the most common gynecologic malignancy in the United States and remains one of the few cancers for which both incidence and mortality continue to increase despite advances in cancer diagnosis and treatment (1–3). Although carboplatin plus paclitaxel remains a standard first-line chemotherapy regimen for advanced and recurrent endometrial cancer, many patients ultimately develop recurrent or chemotherapy-refractory disease. Therefore, identifying mechanism-based therapeutic strategies that enhance chemotherapy response remains a major unmet clinical need. Cisplatin (CDDP) was used in the present study as a mechanistically tractable platinum agent to evaluate platinum sensitization and to facilitate comparison with our prior auranofin (AuR) studies; however, carboplatin-based combinations remain more clinically relevant in the therapeutic context of endometrial cancer.

The Notch signaling pathway regulates cell proliferation, differentiation, stem cell maintenance, and survival, and aberrant NOTCH activation contributes to tumor progression and therapeutic resistance across multiple malignancies (4–14). In endometrial cancer, increasing evidence supports clinically relevant roles for NOTCH signaling, particularly NOTCH2 and NOTCH3 (15). Clinical and molecular studies have linked NOTCH pathway activation to aggressive endometrial cancer states and therapeutic resistance, including studies implicating aberrant NOTCH2 signaling in recurrent endometrioid endometrial cancer (16). In addition, FGFR2-mutant endometrial cancer models exhibit enhanced EGFR and NOTCH signaling, further supporting functional interactions between Notch signaling and oncogenic pathways in endometrial cancer (15, 17–21). Although the role of NOTCH signaling has been extensively investigated in ovarian cancer (22–27), comparatively fewer studies have directly examined NOTCH-directed therapeutic strategies in endometrial cancer.

Although the NOTCH pathway represents an attractive therapeutic target, clinical development of canonical NOTCH inhibitors has been limited by pathway complexity and dose-limiting toxicities (28). γ-secretase inhibitors suppress NOTCH activation through inhibition of Notch intracellular domain (NICD) processing but also affect numerous additional substrates, resulting in dose-limiting gastrointestinal and immune toxicities (29). Similarly, receptor- or ligand-directed approaches have demonstrated limited therapeutic windows in solid tumors (30). More recently, the transcription-complex inhibitor CB-103 demonstrated on-target pharmacodynamics and disease stabilization in early phase I trials (NCT03422679), but objective responses were rare, and its once-daily oral dosing yielded only transient exposures (31, 32). These limitations highlight the need for alternative approaches to perturb oncogenic NOTCH-associated signaling.

We previously identified AuR, an FDA-approved gold-containing compound, using a biochemical screening platform designed to identify small molecules that disrupt RBPJ–DNA interaction, a central downstream event in canonical NOTCH signaling (33). Subsequent studies demonstrated that AuR functions as a noncanonical NOTCH pathway inhibitor by disrupting RBPJ–DNA interaction, reducing RBPJ chromatin occupancy and suppressing NOTCH-dependent transcription in an NICD-driven cellular model (33). More recently, we demonstrated that AuR enhances CDDP efficacy in NOTCH-dependent ovarian cancer models, including patient-derived organoids and xenografts (34). In addition to its effects on NOTCH/RBPJ-associated transcriptional regulation, AuR is also a well-established inhibitor of thioredoxin reductase and can perturb cellular redox homeostasis (35), suggesting that its antitumor activity may reflect integrated effects on RBPJ-associated transcriptional states and oxidative stress adaptation rather than selective inhibition of a single molecular target.

In the present study, we investigated the relationship between NOTCH-associated signaling states and therapeutic response to AuR across a panel of endometrial cancer models. We examined the effects of AuR on RBPJ chromatin occupancy, NOTCH3-dependent therapeutic response, and platinum sensitivity *in vitro* and *in vivo*. Our findings demonstrate that AuR-mediated enhancement of CDDP response is highly context dependent across endometrial cancer models and support further investigation of AuR-based combination strategies in biologically defined endometrial cancer contexts.

## Materials and Methods

### Cell lines and cell culture

RL95-2 (ATCC, CRL-1671, RRID: CVCL_0505), AN3CA (ATCC, HTB-111, RRID: CVCL_0028), and KLE (ATCC, CRL-1622, RRID: CVCL_1329) endometrial cancer cell lines were cultured in RPMI-1640 medium (VWR, cat. #16777-145) supplemented with 10% FBS (VWR, cat. #89510-190), 1% penicillin–streptomycin (VWR, cat. #16777-164), and 2 mmol/L L-glutamine (Gibco, cat. #25030081) at 37°C in a humidified atmosphere containing 5% CO_2_. ARK-1 and ARK-2 cells were obtained from Dr. Alessandro D. Santin, whereas AN3CA, RL95-2, and KLE cells were obtained from K.K. Leslie. Cell lines were routinely monitored for morphology and growth characteristics and were periodically tested for *Mycoplasma* contamination using the InvivoGen MycoStrip Mycoplasma Detection Kit. Short tandem repeat authentication was performed by ATCC (cat. #135-XV-10).

### Protein quantification and immunoblotting

Cells were lysed in 1× SDS sample buffer containing 2.5% SDS, 125 mmol/L Tris-HCl (pH 6.8), and 20% glycerol, without bromophenol blue or dithiothreitol. Lysates were sonicated using a Branson Sonifier 101-135-126 at 25% amplitude for 60 seconds. Protein concentrations were quantified using the Pierce BCA Protein Assay Kit (Thermo Fisher Scientific, cat. #23225). Dithiothreitol and bromophenol blue were subsequently added to the lysates. Equal amounts of protein (10–20 μg per lane) were resolved on 4% to 12% Bis-Tris SurePAGE gels in 1x MOPS SDS running buffer (GenScript, cat. #M00653). Immunoblots were developed using either SuperSignal West Pico PLUS Chemiluminescent Substrate (Thermo Fisher Scientific, cat. #34083) or WesternBright Quantum HRP substrate (Advansta, cat. #K-12042-D10) and imaged using a Konica Processor SRX-101A or a LI-COR Odyssey Fc Imaging System.

### Antibodies

Rabbit polyclonal anti-NOTCH3 antibody was obtained from ABclonal Technology (cat. #A13522, RRID: AB_2760384; 1:2,000). Monoclonal anti-GAPDH antibody was from EMD Millipore (cat. #MAB374, RRID: AB_2107445; 1:10,000). Horseradish peroxidase–conjugated secondary antibodies from Cell Signaling Technology included goat anti–rabbit IgG (cat. #7074, RRID: AB_2099233; 1:3,000) and horse anti–mouse IgG (cat. #7076, RRID: AB_330924; 1:6,000).

### Chromatin immunoprecipitation

Chromatin immunoprecipitation (ChIP) assays were performed as previously described (33, 34, 36, 37). Briefly, approximately 1 × 10^7^ cells were fixed with 1% formaldehyde for 10 minutes and sonicated on ice at 40% amplitude (30 seconds on and 90 seconds off for 24 minutes) using a Branson Sonifier 101-135-126. Immunoprecipitation was performed using a polyclonal anti-RBPJ antibody (FC31; 1:100; ref. 38) and blocked Protein A agarose beads (Thermo Fisher Scientific, cat. #20333). Samples were reverse cross-linked at 65°C for 16 hours. Purified ChIP DNA was analyzed by qRT-PCR using PerfeCTa SYBR Green FastMix, Low ROX (Quantabio) in 384-well format on a QuantStudio 5 Real-Time PCR System (Applied Biosystems). Enrichment of RBPJ at specific genomic regions was normalized to the corresponding input DNA using the ΔCt method (2^−ΔCt^, in which ΔCt = Ct_ChIP − Ct_input). Primer sequences are provided in Supplementary Table S1.

### Lentiviral short hairpin RNA–mediated NOTCH3 knockdown

NOTCH3-targeting short hairpin RNAs (shRNA; GeneCopoeia, cat. #HSH011876-LVRU6GP) and a scrambled control shRNA (GeneCopoeia, cat. #CSHCTR001-LVRU6GP) were used for gene-silencing studies. Lentiviral particles were generated using third-generation packaging plasmids as previously described (34). Cells were infected at approximately 20% confluency, and the medium was replaced after 24 hours. Seventy-two hours after infection, cells were harvested for Western blot analysis of NOTCH3 expression. To establish stable knockdown cell lines, infected cells were selected with puromycin (2 μg/mL) for 7 to 10 days. Stable suppression of NOTCH3 was verified by Western blotting.

### Drug treatment and cell viability measurement

For drug treatment experiments, 25,000 to 50,000 cells were seeded into 96-well plates and treated with varying concentrations of CDDP (MedChemExpress, cat. #HY-17394) and AuR (Cayman Chemical, cat. #15316). After 72 hours of treatment, cell viability was measured using the ATP-Glo Bioluminometric Cell Viability Assay (Biotium, cat. #30020). Luminescence signals were measured using a BioTek Synergy Neo2 plate reader. Selected experiments were independently repeated using crystal violet staining–based viability assays for validation.

Relative viability values were normalized to vehicle-treated controls within each experiment. Dose–response curves were analyzed using nonlinear regression with a four-parameter logistic model in GraphPad Prism software (RRID: SCR_002798). IC_50_ values were calculated from independent biological replicates. In experiments in which dose–response curves exhibited nonparallel behavior and converged at higher drug concentrations, overall drug response was quantified using AUC analysis.

### Xenograft studies

All animals were female NOD/SCID gamma (NSG) immunocompromised mice (RRID: IMSR_JAX:00555), approximately 9 to 11 weeks old at treatment initiation. Each mouse received a single subcutaneous tumor implantation, corresponding to one tumor per mouse and 10 mice per treatment group. Treatment groups were assigned without a formal randomization algorithm. No animals were excluded from the efficacy analyses.

The xenograft studies were conducted by the University of New Mexico Comprehensive Cancer Center Animal Models Shared Resource as a core-facility service. Drug administration and caliper tumor measurements were performed by different core-facility personnel and independently of the investigators conducting the laboratory studies. Caliper measurements were not formally blinded to treatment assignment because cages containing CDDP-treated animals required treatment identification for safe handling.

The AN3CA study was designed as an early-intervention xenograft study, with treatment initiated 3 days after tumor implantation. Pretreatment IVIS imaging was performed on day 3 immediately before treatment initiation to assess baseline tumor burden. Mice received vehicle, AuR (6 mg/kg, i.p., five times weekly), CDDP (1.25 mg/kg, i.p., once weekly), or combined AuR plus CDDP treatment for 25 days.

For the established ARK-1 xenograft study shown in Fig. 6, treatment was initiated 17 days after implantation. In an independent early-treatment ARK-1 study (Supplementary Fig. S7), treatment was initiated 3 days after implantation. Drug dosing schedules were identical to those used in the AN3CA study.

Tumor dimensions were measured twice weekly using calipers, and tumor volumes were calculated using the formula V = (L × W^2^)/2, in which L represents tumor length and W represents tumor width. Tumor-related humane endpoint criteria included tumor volume ≥2,000 mm^3^ or tumor-associated ulceration. Body weight loss exceeding 20% of treatment-start body weight was an additional humane endpoint criterion.

### Ethics statement

All animal studies were approved by the Institutional Animal Care and Use Committee (protocol no. 25-201709-HSC) at the University of New Mexico and were performed in accordance with institutional and NIH guidelines.

### Survival analysis of *NOTCH3* alterations in The Cancer Genome Atlas-Uterine Corpus Endometrial Cancer

Clinical and genomic data for uterine corpus endometrial carcinoma (UCEC) were obtained from The Cancer Genome Atlas (TCGA) through the UCSC Xena Browser. Analyses were restricted to primary tumor samples with one observation per patient. Overall survival (OS) time and status were obtained from TCGA clinical annotations.

For the primary Kaplan–Meier analysis of *NOTCH3* copy number, patients in the upper and lower quartiles of the NOTCH3 copy-number distribution were compared. NOTCH3 RNA expression was analyzed using the same upper-versus-lower quartile stratification. Survival according to *NOTCH3* mutation status was analyzed by comparing tumors with and without NOTCH3 mutations. Kaplan–Meier survival curves were evaluated using log-rank tests.

Robustness analyses included median-based stratification of *NOTCH3* copy number and RNA expression and univariate Cox proportional hazards modeling with *NOTCH3* copy number treated as a continuous variable. To assess whether the upper-quartile *NOTCH3* copy-number association was independent of clinicopathologic and molecular context, multivariable Cox proportional hazards analysis was performed incorporating age, histologic background, tumor grade, and copy number–high molecular subtype. Additional stratified analyses evaluated the *NOTCH3* copy-number association within selected molecular and histologic contexts. Additional details of the robustness analyses are provided in the Supplementary Methods S1.

### Statistical analysis

Statistical analyses were performed using GraphPad Prism. Comparisons between two groups were performed using two-tailed Student *t* tests, as indicated. Comparisons among multiple groups were performed using one-way ANOVA with multiple-comparison correction or Kruskal–Wallis tests with Dunn multiple comparisons, as indicated in the figure legends. Tumor growth curves were analyzed using mixed-effects models with treatment and time as fixed effects. Longitudinal IVIS measurements were analyzed after log_10_ transformation and normalization to pretreatment baseline using two-way repeated-measures ANOVA with Geisser–Greenhouse correction, followed by Holm–Šídák multiple-comparison testing. Kaplan–Meier analyses were evaluated using log-rank (Mantel–Cox) tests. Mice removed for reasons unrelated to a documented tumor-related humane endpoint were censored at the time of removal. *P* values < 0.05 were considered statistically significant.

## Results

### Upper-quartile *NOTCH3* copy number is associated with poorer OS in endometrial cancer

**Figure 1.**
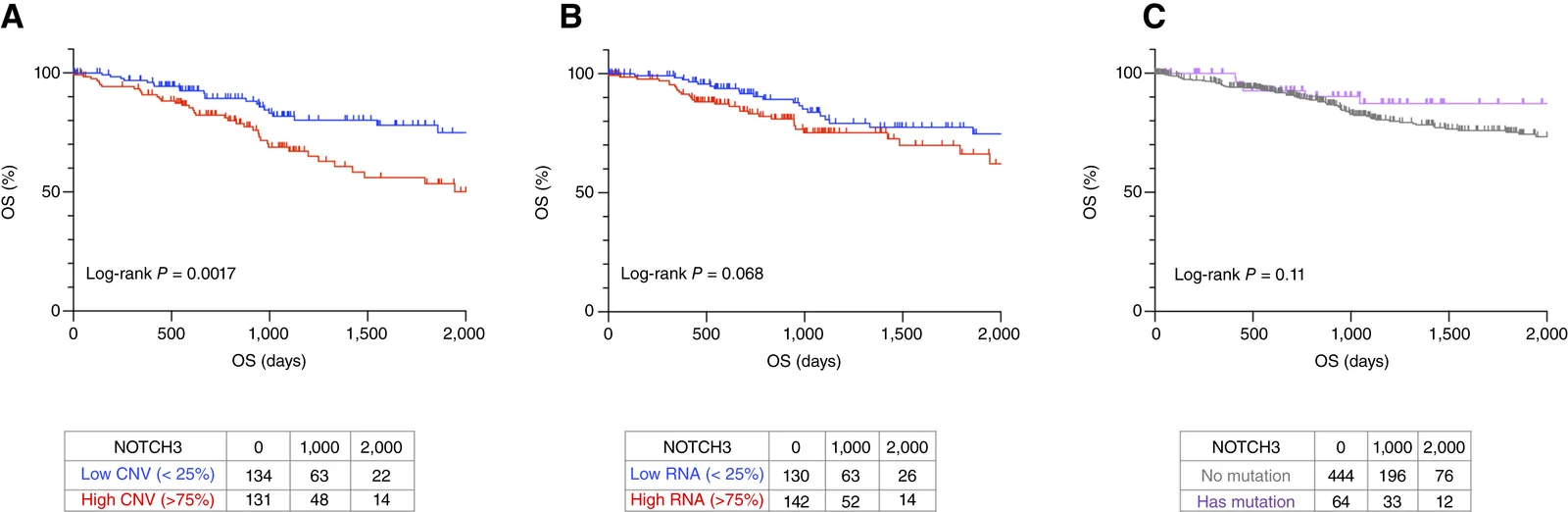
Upper-quartile *NOTCH3* copy number is associated with poorer OS in endometrial cancer. **A,** Kaplan–Meier analysis of OS in the TCGA-UCEC cohort stratified by *NOTCH3* copy number using upper- vs. lower-quartile grouping. Tumors with elevated *NOTCH3* copynumber exhibited significantly reduced OS compared with tumors harboring low copy number. **B,** Kaplan–Meier analysis of OS stratified by NOTCH3 RNA expression using upper- vs. lower-quartile grouping. NOTCH3 RNA expression demonstrated only a modest, nonsignificant association with outcomes. **C,** Kaplan–Meier analysis of OS according to *NOTCH3* mutation status. No significant association between *NOTCH3* mutation status and OS was observed. Log-rank *P* values are indicated on each panel. Tables beneath Kaplan–Meier plots indicate the number of patients at risk at the indicated time points (days). CNV, copy-number variation.

To assess the clinical relevance of *NOTCH3* alterations in endometrial cancer, we analyzed OS in the TCGA-UCEC cohort (Fig. 1). Stratification by *NOTCH3* copy number using segment-level DNA copy values revealed a significant association with patient outcomes. Tumors in the upper quartile of *NOTCH3* copy number exhibited reduced OS compared with tumors in the lower quartile (Fig. 1A; log-rank *P* = 0.0017).

To assess the robustness and independence of this association, we performed additional survival analyses using alternative stratification strategies and clinicopathologic covariates. Median-based stratification of *NOTCH3* copy number was not significantly associated with OS (Supplementary Fig. S1A; log-rank *P* = 0.13), and continuous Cox proportional hazards modeling likewise did not identify a significant linear association between *NOTCH3* copy number and OS [HR, 1.30; 95% confidence interval (CI), 0.62–2.56; *P* = 0.48].

We further evaluated the upper-quartile *NOTCH3* copy-number association using a multivariable Cox model incorporating clinicopathologic and molecular context. After adjustment for age, histologic background, tumor grade, and copy number–high molecular subtype, the association between upper-quartile *NOTCH3* copy number and OS was attenuated and no longer statistically significant (adjusted HR, 1.85; 95% CI, 0.93–3.89; *P* = 0.091). Consistent with this result, upper-versus-lower quartile *NOTCH3* copy-number status was not significantly associated with survival when the analysis was restricted to the copy number–high molecular subgroup. Together, these findings indicate that the adverse survival association observed in the unadjusted quartile analysis is context dependent and may partly reflect the broader copy number–high molecular background rather than an independent prognostic effect of *NOTCH3* copy number alone.

Using the same upper-versus-lower quartile stratification approach, NOTCH3 RNA expression demonstrated only a modest, nonsignificant association with survival (Fig. 1B), suggesting that transcript abundance alone may not adequately capture biologically relevant NOTCH3 pathway activity. Consistent with this observation, median-based stratification of NOTCH3 RNA expression similarly yielded a weak association with outcomes (Supplementary Fig. S1B). Likewise, *NOTCH3* mutation status was not significantly associated with OS (Fig. 1C), consistent with the relatively low frequency and heterogeneous functional consequences of *NOTCH3* mutations in endometrial cancer.

Collectively, these findings indicate that the association between *NOTCH3* copy number and clinical outcomes is most apparent at the upper end of the copy-number distribution and is dependent on molecular context. We therefore next investigated whether NOTCH3-associated signaling contributes to therapeutic vulnerability in endometrial cancer models.

### Endometrial cancer models exhibit heterogeneous molecular backgrounds and therapeutic response profiles

To place the experimental models within the modern molecular–pathologic framework of endometrial cancer, we compiled their reported histologic origins and major genomic features (Supplementary Table S2). The panel spans biologically distinct endometrial cancer contexts, including mismatch repair–deficient (MMRd) and p53-abnormal/copy number–high–like models. AN3CA is an undifferentiated/poorly differentiated endometrial cancer model with an MMRd molecular background, whereas ARK-1 and ARK-2 are uterine serous carcinoma models with TP53 alterations, consistent with a p53-abnormal/copy number–high–like context. RL95-2 exhibits a mixed molecular profile with reported copy number–high features, whereas KLE carries a pathogenic TP53 alteration consistent with a p53-abnormal–like background. These molecular–context assignments are approximate and are intended to provide biological context rather than the formal TCGA classification of the cell lines.

**Figure 2.**
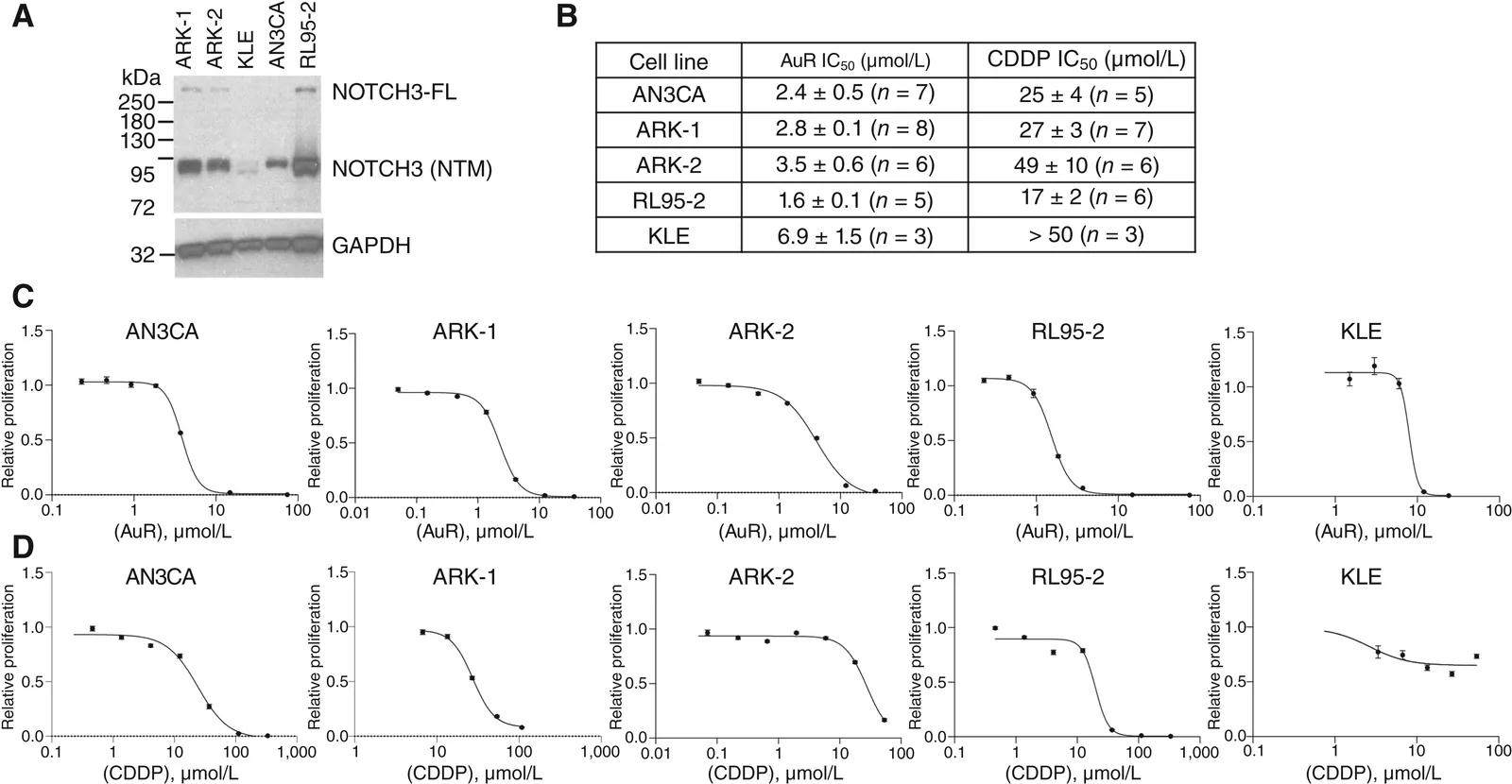
NOTCH3 protein expression and sensitivity to AuR and CDDP in endometrial cancer cell lines. **A,** Representative Western blot analysis of full-length NOTCH3 (NOTCH3-FL) and a cleaved NOTCH3/NTM-reactive species in endometrial cancer cell lines. GAPDH served as a loading control. **B,** Summary of AuR and CDDP IC_50_ values across endometrial cancer cell lines. IC_50_ values are presented as mean ± SEM from independent biological replicates. **C,** Dose–response analysis of AuR sensitivity in endometrial cancer cell lines. **D,** Dose–response analysis of CDDP sensitivity in endometrial cancer cell lines. Dose–response curves in **C** and **D** were fitted using a four-parameter logistic regression model.

To evaluate NOTCH3 protein expression and therapeutic response across endometrial cancer models, we characterized a panel of endometrial cancer cell lines for *NOTCH3*-derived protein species and sensitivity to AuR and CDDP (Fig. 2). Representative Western blot analysis demonstrated heterogeneous NOTCH3 protein patterns across the models examined (Fig. 2A). A prominent cleaved NOTCH3/NTM-reactive species was detected in several cell lines, whereas KLE cells showed comparatively weak signal. Because the immunoblot shown represents a single cross–cell line comparison, we did not quantitatively rank NOTCH3 abundance among the models.

To quantitatively compare therapeutic responses across models, IC_50_ values for AuR and CDDP were determined following 72-hour treatment (Fig. 2B). RL95-2 and AN3CA cells were among the most sensitive to AuR, whereas KLE cells exhibited relative resistance. Similarly, KLE cells displayed marked resistance to CDDP compared with the other endometrial cancer models examined. Representative dose–response curves for AuR and CDDP are shown in Fig. 2C and D, respectively. *NOTCH3*-derived protein patterns and therapeutic responsiveness varied across the endometrial cancer models examined. Notably, KLE cells exhibited comparatively weak NOTCH3/NTM signal while remaining relatively resistant to both AuR and CDDP. These observations prompted us to investigate directly whether NOTCH3-associated signaling contributes to therapeutic vulnerability across endometrial cancer models.

### AuR suppresses RBPJ occupancy and potentiates CDDP response in endometrial cancer cells

**Figure 3.**
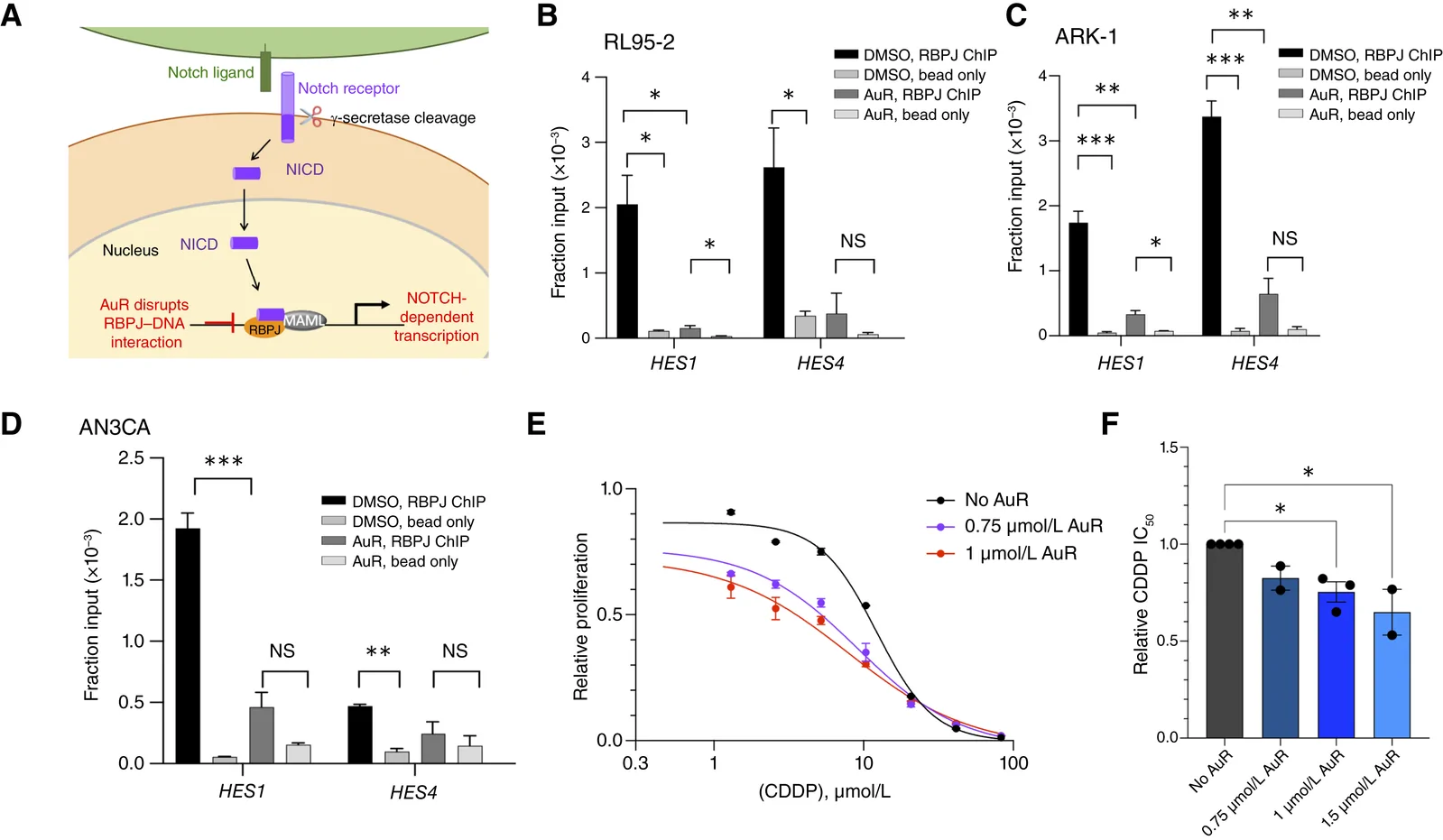
AuR suppresses RBPJ occupancy and enhances CDDP response in endometrial cancer cells. **A,** Schematic working model illustrating canonical NOTCH/RBPJ signaling and the effect of AuR on RBPJ–DNA interaction. Following ligand-dependent NOTCH activation and γ-secretase cleavage, NICD translocates to the nucleus and forms a transcriptional activation complex with RBPJ and MAML at NOTCH-responsive loci. AuR disrupts RBPJ–DNA interaction and reduces RBPJ chromatin occupancy. **B–D,** Representative RBPJ ChIP assays in RL95-2 (**B**), ARK-1 (**C**), and AN3CA (**D**) cells following treatment with DMSO or 2.5 μmol/L AuR. RBPJ occupancy at the *HES1* and *HES4* loci was measured by qPCR and normalized to input chromatin. Bead-only controls were included to assess nonspecific background enrichment. **E,** Representative nonlinear regression curves from one experiment showing CDDP response in AN3CA cells treated with increasing concentrations of CDDP in the absence or presence of 0.75 or 1 μmol/L AuR. Relative proliferation was normalized to untreated controls. **F,** Replicate-level quantification of relative CDDP IC_50_ values from independent biological replicates across AuR concentrations, including 0.75, 1, and 1.5 μmol/L AuR conditions. IC_50_ values were normalized to the no-AuR condition. Data are presented as mean ± SEM. Statistical significance in **F** was determined using one-way ANOVA with multiple-comparison correction. NS, not significant; *, *P* < 0.05; **, *P* < 0.01; ***, *P* < 0.001.

Given the heterogeneous NOTCH3-derived protein patterns and AuR responsiveness observed across endometrial cancer models (Fig. 2), we next examined whether AuR alters RBPJ occupancy at canonical NOTCH target loci and modulates platinum response. A schematic model of the proposed mechanism is shown in Fig. 3A. In RL95-2 cells, strong RBPJ enrichment above bead-only background was detected at both the *HES1* and *HES4* loci under basal conditions (Fig. 3B). Following AuR treatment, RBPJ enrichment at *HES1* was markedly reduced, whereas residual enrichment at *HES4* was diminished and no longer significantly above bead-only background. Similarly, ARK-1 cells demonstrated robust basal RBPJ enrichment at both *HES1* and *HES4* loci (Fig. 3C). AuR treatment reduced RBPJ occupancy at both loci. AN3CA cells also demonstrated detectable RBPJ enrichment at *HES1* and *HES4*, although the magnitude of enrichment was lower than that observed in RL95-2 and ARK-1 cells (Fig. 3D). AuR treatment reduced RBPJ occupancy at both loci. In separate qRT-PCR experiments, treatment with 1.25 or 2.5 μmol/L AuR for 24 hours did not significantly alter *HES1* or *HES4* transcript levels in AN3CA or ARK-1 cells (Supplementary Table S3).

To determine whether AuR alters platinum response, AN3CA cells were treated with increasing concentrations of CDDP in the presence of subcytotoxic concentrations of AuR. As shown in Fig. 3E, AuR treatment produced a dose-dependent leftward shift in CDDP response relative to cells treated with CDDP alone. The CDDP response curves generated in the presence of AuR maintained similar overall shapes and slopes, permitting reliable IC_50_ analysis. Quantification of replicate-level IC_50_ values demonstrated that AuR significantly reduced relative CDDP IC_50_ values in a dose-dependent manner (Fig. 3F).

To determine whether AuR-mediated enhancement of CDDP response extends beyond AN3CA cells, additional combination studies were performed in RL95-2 cells (Supplementary Fig. S2). AuR treatment produced a concentration-dependent leftward shift in CDDP dose–response curves. Quantification of replicate-level IC_50_ values demonstrated significantly reduced relative CDDP IC_50_ values in the presence of both 1 and 2 μmol/L AuR. These findings further support the ability of AuR to potentiate platinum response in selected endometrial cancer models.

### AN3CA exhibits a distinct NOTCH3-dependent therapeutic response

**Figure 4.**
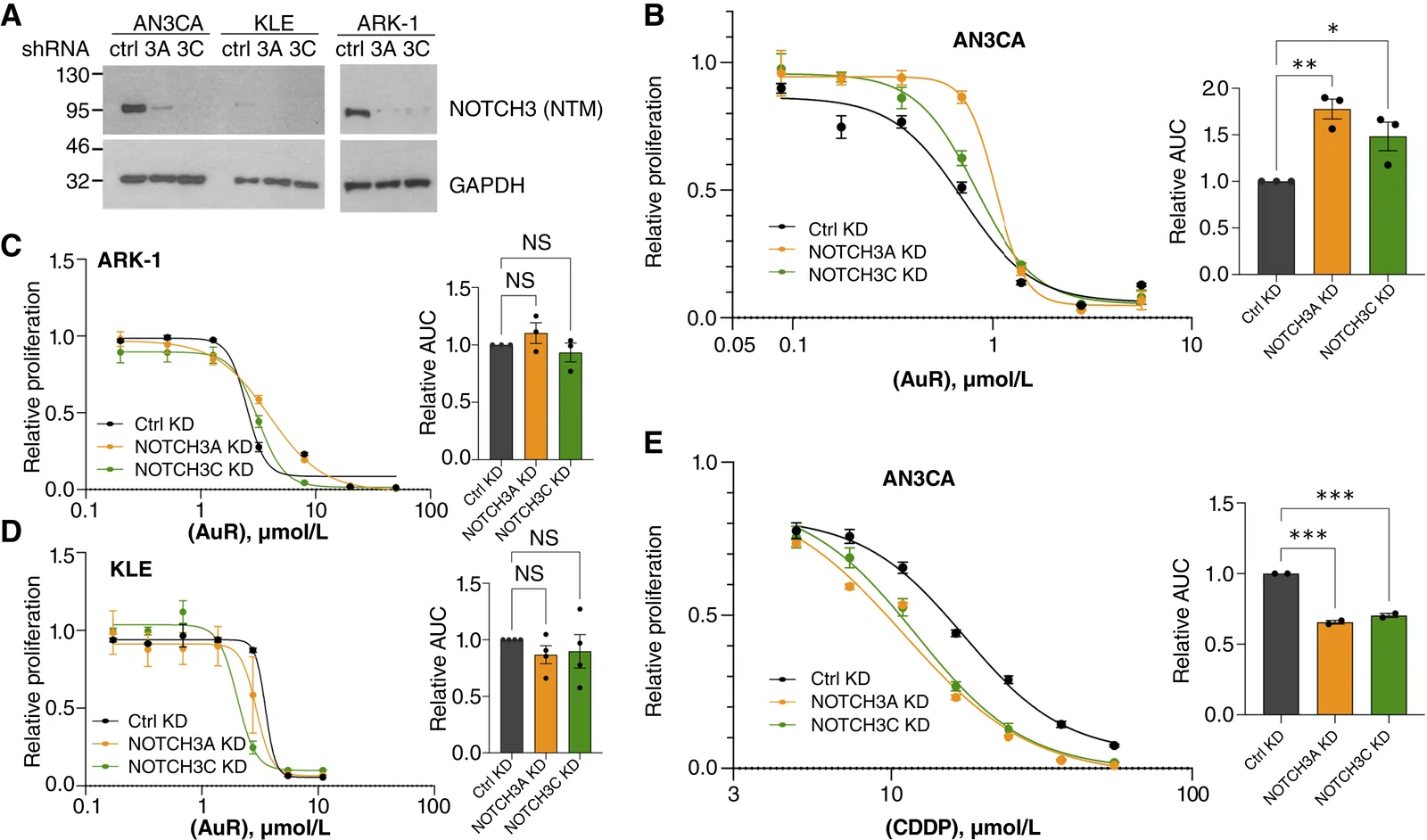
NOTCH3 depletion alters AuR and CDDP response in a context-dependent manner across endometrial cancer models. **A,** Western blot validation of NOTCH3 knockdown in AN3CA, KLE, and ARK-1 cells using independent shRNAs targeting NOTCH3 (3A and 3C). GAPDH served as a loading control. **B–D,** Dose–response analysis of AuR response in Ctrl KD, NOTCH3A-KD, and NOTCH3C-KD cells. Representative nonlinear regression curves are shown (left), with replicate-level AUC quantification (right). **E,** Dose–response analysis of CDDP response in AN3CA cells following NOTCH3 depletion. Representative nonlinear regression curves are shown (left), with replicate-level AUC quantification (right). Relative proliferation values were normalized to vehicle-treated controls. Data are presented as mean ± SEM. AUC values were calculated from replicate dose–response curves. Statistical significance was determined using one-way ANOVA with multiple-comparison correction. *, *P* < 0.05; **, *P* < 0.01; ***, *P* < 0.001; NS, not significant. Ctrl, control; KD, knockdown.

To determine whether NOTCH3 directly contributes to AuR response in endometrial cancer cells, we generated stable NOTCH3-knockdown models using two independent shRNAs targeting NOTCH3 (3A and 3C knockdown) in AN3CA, KLE, and ARK-1 cells (Fig. 4A). Western blot analysis confirmed efficient depletion of the cleaved NOTCH3/NTM species in AN3CA and ARK-1 cells, whereas KLE cells exhibited comparatively weak baseline signal.

We next examined the effect of NOTCH3 depletion on AuR response. In AN3CA cells, knockdown with either NOTCH3-targeting shRNA reproducibly reduced AuR sensitivity relative to control knockdown cells (Fig. 4B). Because the dose–response curves converged at higher AuR concentrations, overall AuR responsiveness was quantified using AUC analysis, which demonstrated significantly increased AUC following NOTCH3 depletion. These findings indicate that NOTCH3 contributes to AuR responsiveness in AN3CA cells.

In contrast, NOTCH3 depletion produced minimal or nonsignificant effects on AuR response in ARK-1 and KLE cells (Fig. 4C and D). Additional studies in RL95-2 and ARK-2 cells likewise demonstrated little evidence of NOTCH3-dependent AuR responsiveness (Supplementary Fig. S3). Thus, the effect of NOTCH3 depletion on AuR response was not shared uniformly across endometrial cancer models.

Because NOTCH3 signaling has been implicated in platinum resistance, we next examined whether NOTCH3 depletion alters CDDP response. In AN3CA cells, knockdown with either NOTCH3-targeting shRNA significantly enhanced CDDP responsiveness, producing a pronounced leftward shift in the CDDP dose–response curves, and reduced AUC values (Fig. 4E). In contrast, NOTCH3 depletion did not significantly alter CDDP response in ARK-1 or ARK-2 cells (Supplementary Fig. S3).

Together, these findings identify AN3CA as a distinct NOTCH3-dependent endometrial cancer context in which NOTCH3 contributes both to AuR responsiveness and to platinum resistance. The absence of comparable NOTCH3 dependence in the other endometrial cancer models examined supports a context-dependent, rather than universal, relationship between NOTCH3 dependence and AuR responsiveness.

### AuR enhances CDDP efficacy in AN3CA xenografts

**Figure 5.**
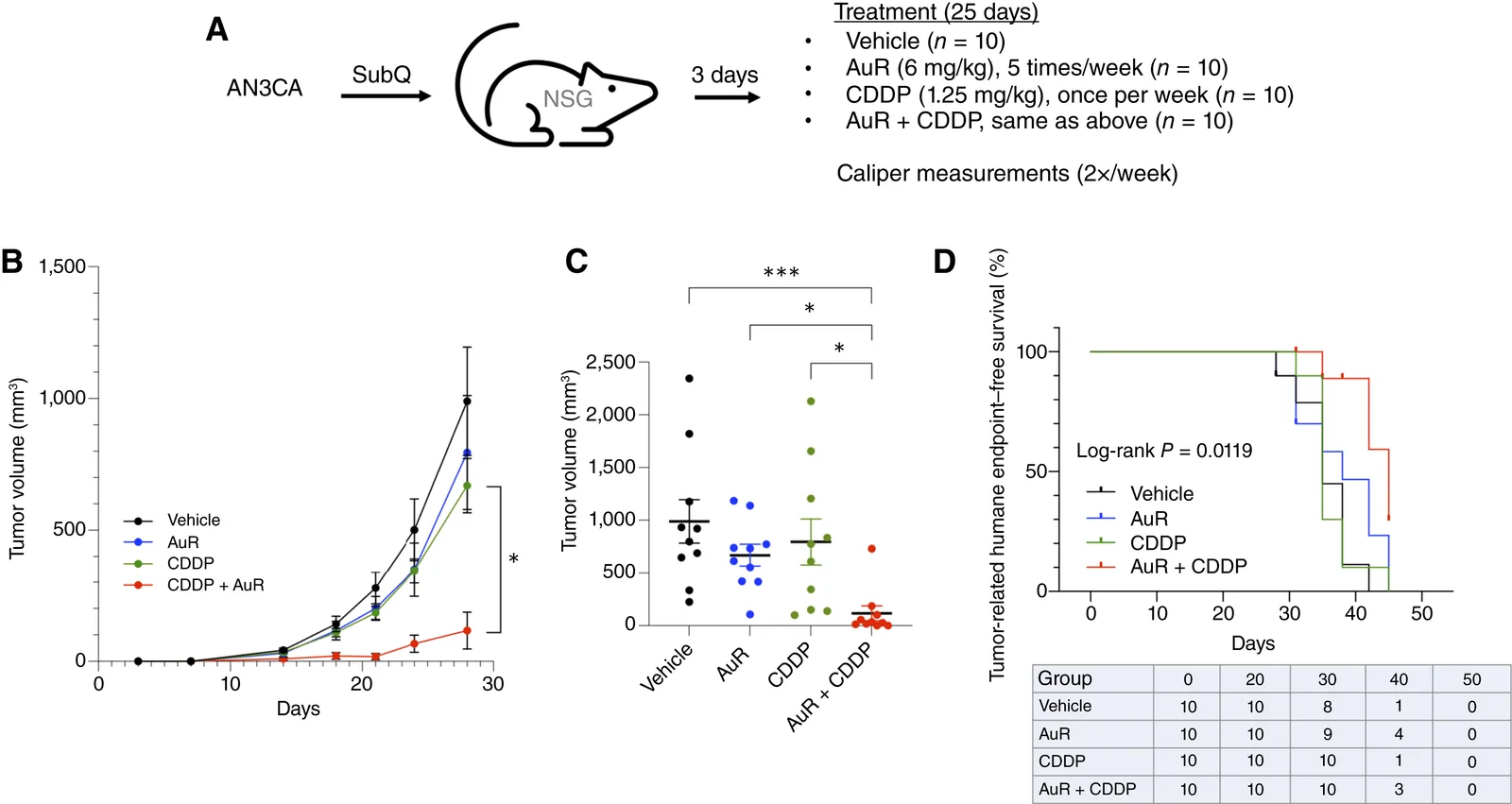
AuR enhances CDDP efficacy in AN3CA xenografts. **A,** Schematic of the AN3CA subcutaneous xenograft study design. Tumor growth was monitored by caliper measurements. **B,** Tumor growth curves showing mean ± SEM tumor volume over time. **C,** Endpoint tumor volumes on day 28. Each dot represents an individual mouse. **D,** Kaplan–Meier analysis of time to tumor-related humane endpoint, defined as tumor volume ≥2,000 mm^3^ or tumor-associated ulceration. Censoring marks indicate mice removed for reasons unrelated to a documented tumor endpoint, and numbers at risk are shown below the survival curves. The overall difference among treatment groups was significant by a log-rank (Mantel–Cox) test (*P* = 0.0119). Pairwise comparison demonstrated significantly prolonged endpoint-free survival with AuR + CDDP compared with CDDP alone (log-rank *P* = 0.0044). Statistical significance was determined using mixed-effects model analysis (**B**), Kruskal–Wallis test with Dunn multiple-comparison correction (**C**), and log-rank testing (**D**). *, *P* < 0.05; ***, *P* < 0.001. SubQ, subcutaneous.

To determine whether the CDDP-potentiating effects of AuR observed *in vitro* extend to an *in vivo* setting, we performed an early-intervention AN3CA subcutaneous xenograft study in NSG mice, with treatment initiated 3 days after tumor implantation (Fig. 5A). Mice received vehicle, AuR alone, CDDP alone, or combined AuR plus CDDP treatment for 25 days. Vehicle-treated tumors displayed continuous expansion throughout the study period (Fig. 5B). AuR monotherapy produced modest growth delay, whereas CDDP treatment partially constrained tumor progression. In contrast, combined AuR plus CDDP treatment strongly inhibited tumor expansion relative to vehicle and either monotherapy. Endpoint analysis demonstrated markedly lower tumor burden in mice receiving combination therapy compared with vehicle-, AuR-, and CDDP-treated cohorts (Fig. 5C). Kaplan–Meier analysis of time-to-tumor–related humane endpoint demonstrated a significant overall difference among treatment groups (log-rank *P* = 0.0119), with AuR + CDDP significantly prolonging endpoint-free survival compared with CDDP alone (*P* = 0.0044; Fig. 5D). Collectively, these findings demonstrate that AuR potentiates CDDP antitumor activity in AN3CA xenografts.

Because AN3CA cells expressed Luc2, longitudinal IVIS imaging provided an independent assessment of tumor burden. Pretreatment imaging on day 3 confirmed detectable tumor-associated bioluminescence before treatment initiation. Analysis of baseline-normalized IVIS measurements demonstrated a significant overall treatment effect (*P* = 0.0003; Supplementary Fig. S4). AuR + CDDP produced significantly lower IVIS tumor burden than CDDP alone at multiple posttreatment time points, and cumulative IVIS tumor burden over the treatment period was significantly lower with AuR + CDDP than with CDDP alone (Holm–Šídák adjusted *P* = 0.0045). These findings independently corroborate the caliper-based therapeutic response.

To assess treatment tolerability, body weight was monitored throughout the AN3CA xenograft study and normalized to treatment-start weight (Supplementary Fig. S5). Mice receiving AuR + CDDP exhibited greater body weight reduction than the other treatment groups, consistent with a measurable treatment-related effect. However, no mouse in any treatment group fell below the prespecified humane-endpoint threshold of 80% of treatment-start body weight during the treatment period. Thus, the combination produced detectable body weight effects but did not result in body weight loss reaching the prespecified humane-endpoint threshold under the dosing schedule used.

### AuR-mediated enhancement of CDDP response is not observed in ARK-1 xenografts

**Figure 6.**
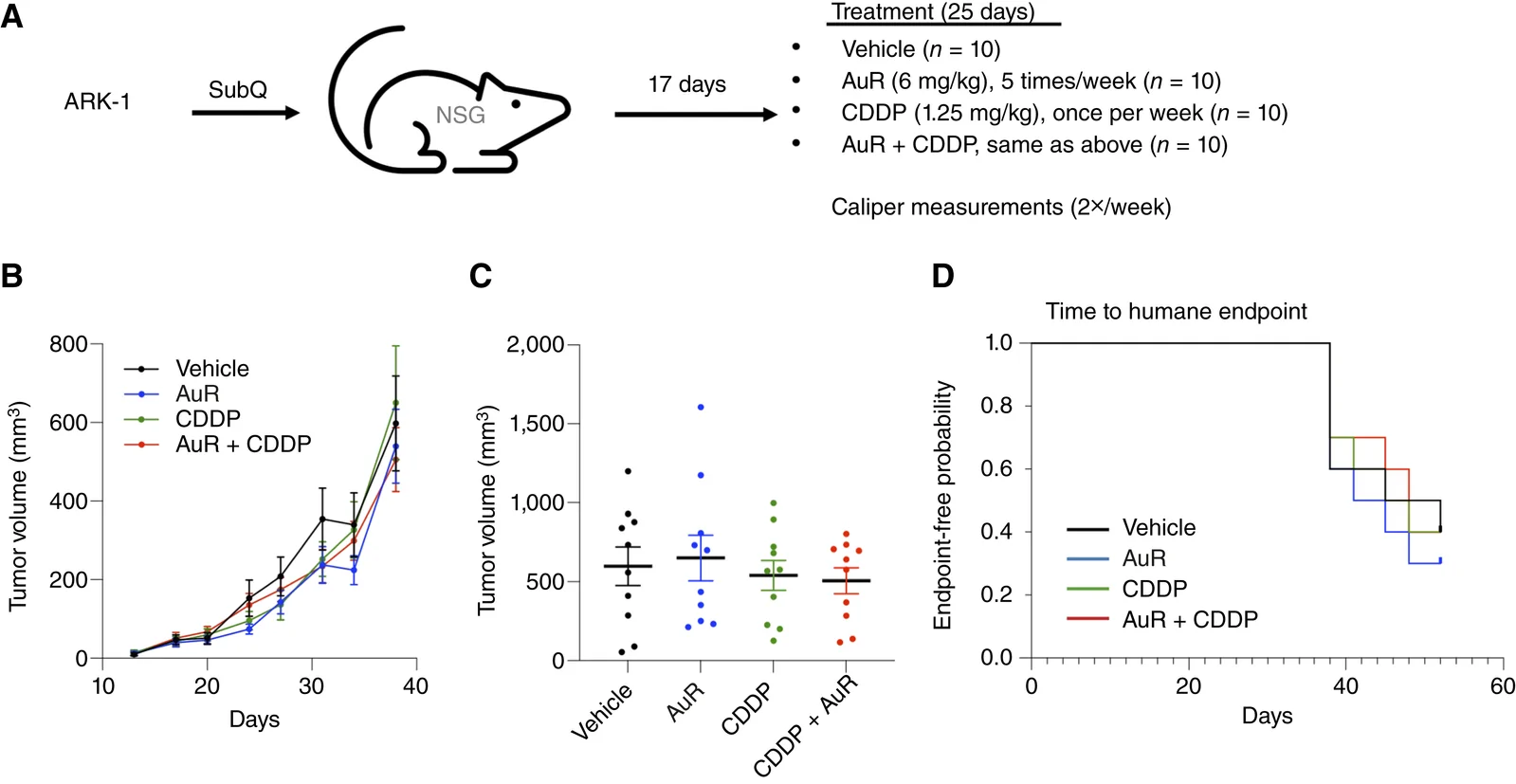
ARK-1 xenografts exhibit limited response to combined AuR and CDDP treatment. **A,** Experimental design. ARK-1 cells were implanted subcutaneously (SubQ) into NSG mice. Treatment was initiated 17 days after implantation and continued for 25 days. Mice received vehicle, AuR (6 mg/kg, i.p., five times per week), CDDP (1.25 mg/kg, i.p., once weekly), or the combination of AuR and CDDP (*n* = 10 mice per group). Tumor volumes were measured twice weekly by caliper. **B,** Tumor growth curves during treatment. Mean tumor volume ± SEM is shown for each treatment group. **C,** Tumor volumes at day 38 (end of treatment). Each symbol represents an individual tumor; horizontal lines indicate mean ± SEM. **D,** Kaplan–Meier analysis of time to humane tumor endpoint. Statistical significance was determined using mixed-effects model analysis (**B**), Kruskal–Wallis test with Dunn’ multiple-comparison correction (**C**), and log-rank test (**D**).

Given the detectable NOTCH3/NTM signal and robust RBPJ occupancy observed in ARK-1 cells (Figs. 2 and 3), we next evaluated whether AuR could enhance CDDP efficacy *in vivo* using this model. ARK-1 cells were implanted subcutaneously into NSG mice, and treatment was initiated 17 days after implantation, after tumors had become established (Fig. 6A–D).

Vehicle-treated tumors progressively increased in size throughout the study (Fig. 6B). AuR monotherapy produced only modest effects on tumor growth, whereas CDDP partially suppressed tumor progression. However, combined AuR plus CDDP treatment did not further reduce tumor growth relative to CDDP alone. Consistent with these findings, endpoint tumor volumes were not significantly different between the CDDP and combination treatment groups (Fig. 6C). Similarly, Kaplan–Meier analysis revealed no clear improvement in time-to-humane-tumor endpoint with combined treatment compared with CDDP monotherapy (Fig. 6D).

Body weight was monitored during the established ARK-1 xenograft study to assess treatment tolerability (Supplementary Fig. S6). Body weight remained comparatively stable during treatment, and no mouse in any treatment group fell below the prespecified humane-endpoint threshold of 80% of treatment-start body weight. These findings indicate that severe body weight loss was not observed under the dosing schedule used.

To evaluate whether treatment timing influenced therapeutic response, an independent ARK-1 xenograft study was initiated 3 days after tumor implantation (Supplementary Fig. S7). Despite differences in tumor growth kinetics, combined AuR plus CDDP treatment again failed to demonstrate a reproducible improvement over CDDP alone. Together, these findings indicate that AuR-mediated enhancement of CDDP response is context dependent and may not be predicted solely by detectable NOTCH3/NTM signal or AuR-mediated suppression of RBPJ occupancy.

## Discussion

In the present study, we demonstrate that upper-quartile *NOTCH3* copy number is associated with adverse clinical outcomes in endometrial cancer, that AuR suppresses RBPJ occupancy at canonical NOTCH target loci, and that AuR potentiates CDDP response in selected endometrial cancer models both *in vitro* and *in vivo*. At the same time, our findings reveal substantial biological heterogeneity across endometrial cancer models, indicating that NOTCH-associated therapeutic vulnerabilities are highly context dependent.

Although upper-quartile *NOTCH3* copy number was associated with poorer OS in unadjusted analysis, this relationship was not retained using median-based stratification, continuous Cox modeling, or multivariable adjustment for clinicopathologic and molecular context. In particular, attenuation of the association after accounting for copy number–high molecular subtype suggests that elevated *NOTCH3* copy number may partly mark a broader aggressive genomic state rather than function as an independent prognostic biomarker. Accordingly, we interpret the TCGA finding as a context-dependent clinical association that is most apparent at the upper end of the *NOTCH3* copy-number distribution and requires validation in independent molecularly annotated cohorts.

A further limitation of the copy-number analysis is that *NOTCH3* resides within the broader 19p13.12 amplicon. This gene-dense region may undergo regional amplification together with neighboring genes. Prior characterization of the 19p13.12 amplicon identified *NOTCH3* as a prominent candidate driver but also demonstrated that multiple genes can be co-amplified within this interval. Accordingly, the survival association observed in TCGA-UCEC cannot be attributed uniquely to *NOTCH3* copy-number gain and may partly reflect broader amplicon or copy number–high tumor biology. Future biomarker studies incorporating validated functional NOTCH-pathway readouts in addition to genomic copy-number status will be required to determine whether combined genomic and pathway-state measurements improve predictive value.

Mechanistically, our findings support a model in which AuR-mediated platinum sensitization reflects context-dependent contributions from NOTCH/RBPJ-associated signaling and cellular redox adaptation. AuR treatment reduced RBPJ occupancy at canonical NOTCH target loci, including *HES1* and *HES4*, across multiple endometrial cancer models, extending our prior identification of AuR as a noncanonical NOTCH pathway inhibitor that disrupts RBPJ–DNA interaction. In separate qRT-PCR experiments, however, AuR did not significantly alter steady-state *HES1* or *HES4* transcript levels in AN3CA or ARK-1 cells under the conditions tested. Together with our prior demonstration that AuR reduced *HES1* and *HEY1* expression in an NICD-driven cellular model (33), these findings indicate that the downstream transcriptional consequences of disrupting RBPJ–DNA interaction are context dependent.

Several observations further indicate that disruption of canonical NOTCH/RBPJ signaling alone does not fully explain the biological activity of AuR in endometrial cancer. In AN3CA cells, two independent NOTCH3-targeting shRNAs reduced AuR sensitivity while enhancing CDDP sensitivity, providing genetic evidence that NOTCH3 contributes to both AuR responsiveness and platinum resistance in this model. In contrast, NOTCH3 depletion produced minimal or nonsignificant effects on therapeutic response in the other endometrial cancer models examined, and reduced RBPJ occupancy did not uniformly predict either *HES1*/*HES4* expression or therapeutic response. Moreover, AuR retained substantial cytotoxic activity in KLE cells despite comparatively weak NOTCH3/NTM signal. Thus, AuR-mediated chemosensitization is best viewed as a context-dependent, multimechanistic process rather than as a uniform consequence of canonical NOTCH inhibition alone.

One potential explanation for this broader activity is the well-established role of AuR as an inhibitor of thioredoxin reductase and cellular redox homeostasis (39). Because platinum agents induce oxidative and metabolic stress during chemotherapy exposure, simultaneous disruption of redox adaptation pathways could cooperate with perturbation of RBPJ-associated transcriptional programs to lower the threshold for chemotherapy-induced cytotoxicity. Such dual targeting may distinguish AuR from pathway-restricted canonical NOTCH inhibitors. Emerging evidence further supports functional cross-talk between RBPJ-associated transcriptional states and oxidative stress pathways. Previous studies have demonstrated that NOTCH/RBPJ signaling can regulate NRF2-associated cytoprotective responses and cellular antioxidant adaptation (40), whereas ROS-dependent NRF2–NOTCH interactions contribute to adaptive cellular signaling states (41). Consistent with these observations, AuR has also been reported to modulate NRF2/Keap1-associated stress adaptation pathways during chemotherapy sensitization (42). Together, these observations support the possibility that AuR response reflects integrated NOTCH/redox signaling states rather than isolated inhibition of canonical NOTCH target genes.

Importantly, AuR monotherapy produced only modest antitumor activity in most settings (Figs. 5 and 6), whereas substantially greater effects were observed when AuR was combined with CDDP (Fig. 5). Moreover, potentiation of platinum response was achieved using subcytotoxic AuR concentrations *in vitro*, raising the possibility that lower-dose combination strategies may provide therapeutic benefit while potentially minimizing toxicities associated with more extensive pathway inhibition. This concept may be particularly relevant given the dose-limiting gastrointestinal toxicities historically associated with pan-NOTCH inhibition using γ-secretase inhibitors.

The body weight analyses provide an initial assessment of treatment tolerability in both xenograft models (Supplementary Figs. S5 and S6). In AN3CA xenografts, combined AuR plus CDDP produced clear antitumor benefit but was associated with greater body weight reduction than the other treatment groups. In the established ARK-1 model, body weight remained comparatively stable during treatment. Importantly, no mouse in either study reached the prespecified humane-endpoint threshold of 80% of treatment-start body weight. These findings indicate that the combination produced detectable, model-dependent tolerability effects but did not result in body weight loss reaching the humane-endpoint threshold under the dosing schedules used. Because body weight is an indirect safety measure and can be influenced by tumor burden, future studies will require formal dose optimization, clinical chemistry, tissue histopathology, and expanded toxicity assessment to define the therapeutic index of AuR–platinum combinations.

The relationship between NOTCH3 activity and platinum response was particularly notable in AN3CA models. AN3CA is an undifferentiated/poorly differentiated MMRd endometrial cancer model with intermediate platinum responsiveness. Genetic suppression of NOTCH3 significantly enhanced CDDP responsiveness, and pharmacologic AuR treatment similarly potentiated CDDP activity both *in vitro* and *in vivo* (Figs. 4 and 5). Importantly, the enhancement observed with AuR was not solely attributable to baseline cytotoxicity, as combination treatment additionally shifted CDDP dose–response relationships toward lower CDDP concentrations. These findings are consistent with prior studies linking NOTCH signaling to platinum resistance and further support the concept that modulation of NOTCH-associated transcriptional programs can alter chemotherapy response thresholds in selected endometrial cancer contexts (23, 25, 34, 43–46). Although reduced RBPJ occupancy supports disruption of canonical NOTCH-associated chromatin engagement, future studies will be required to define the broader transcriptional consequences of AuR treatment in endometrial cancer cells.

The contrasting responses of AN3CA and ARK-1 should also be considered in the context of their distinct histologic and molecular backgrounds. AN3CA represents an undifferentiated/poorly differentiated endometrial cancer model with an MMRd molecular background, whereas ARK-1 is a uterine serous carcinoma model with a TP53 alteration, consistent with a p53-abnormal/copy number–high–like context (Supplementary Table S2). These differences raise the possibility that AuR-mediated platinum sensitization reflects broader context-specific signaling dependencies in addition to variation in NOTCH/RBPJ- or redox-associated pathways. Because the present study includes a limited number of models within each molecular context, these observations should be regarded as hypothesis-generating rather than as evidence for a definitive molecular subtype biomarker.

In contrast, ARK-1 xenografts demonstrated limited benefit from combined AuR plus CDDP treatment despite detectable NOTCH3/NTM signal and suppression of RBPJ occupancy following AuR exposure. An independent ARK-1 study initiated shortly after tumor implantation similarly failed to demonstrate a reproducible enhancement of CDDP efficacy by AuR. Importantly, AN3CA was the only endometrial cancer model in which NOTCH3 depletion reproducibly altered both AuR responsiveness and CDDP sensitivity, identifying AN3CA as a distinct NOTCH3-dependent context. These findings indicate that the NOTCH3-associated signaling state contributes to both AuR responsiveness and platinum resistance in AN3CA, but we do not interpret NOTCH3 depletion as a genetic phenocopy of AuR treatment. In contrast, NOTCH3 depletion produced minimal or nonsignificant effects on therapeutic response in ARK-1, KLE, RL95-2, and ARK-2 cells. Together, these findings indicate that NOTCH3 dependence is not a universal feature of endometrial cancer and highlight the context-dependent nature of AuR responsiveness. Such heterogeneity likely reflects differences in both molecular background and signaling dependencies among endometrial cancer tumors and suggests that successful deployment of AuR-based therapeutic strategies may require biologically informed patient stratification rather than reliance on a single-pathway biomarker.

Several limitations of the present study should be acknowledged. Although our findings support a role for NOTCH/RBPJ-associated signaling in modulating platinum response, the precise molecular mechanisms linking AuR treatment to altered chemotherapy sensitivity remain incompletely defined. Reduced RBPJ occupancy supports disruption of RBPJ-associated chromatin engagement but does not establish that RBPJ inhibition is the sole mechanism of AuR-mediated chemosensitization. In addition, the AN3CA xenograft study used an early-intervention design rather than a conventional established-tumor model, and the present study evaluated CDDP rather than carboplatin, which is more commonly incorporated into contemporary endometrial cancer chemotherapy regimens. Future studies using established and patient-derived endometrial cancer models, together with validation using carboplatin and carboplatin/paclitaxel-based regimens, will be important for defining clinically relevant response states and determining the translational potential of this strategy.

In summary, our findings demonstrate that AuR suppresses RBPJ occupancy at canonical NOTCH target loci and potentiates platinum response in selected endometrial cancer contexts. Collectively, the data support a model in which AuR alters chemotherapy responsiveness through context-dependent contributions from NOTCH-associated and redox-associated survival pathways and warrant further investigation of AuR-based combination strategies in biologically defined endometrial cancer contexts.

## Supplementary Material

Supplementary Data Sup. Methods

Supplementary Table S1 Primers used in qPCR

Supplementary Table S2 Histologic and molecular characteristics of endometrial cancer cell-line models

Supplementary Table S3 RT-qPCR analysis of HES1 and HES4 expression following auranofin treatment in AN3CA and ARK-1 cells.

Supplementary Fig. S1 Robustness analysis of NOTCH3 survival associations.

Supplementary Fig. S2 AuR enhances CDDP response in additional endometrial cancer cell models.

Supplementary Fig. S3 NOTCH3 depletion produces minimal effects on AuR and CDDP responsiveness in additional endometrial cancer models.

Supplementary Fig. S4 Longitudinal IVIS analysis of AN3CA-Luc2 xenografts confirms enhanced tumor control with combined AuR and CDDP treatment.

Supplementary Fig. S5 Body-weight monitoring in AN3CA xenograft-bearing mice during treatment.

Supplementary Fig. S6 Body-weight monitoring in ARK-1 xenograft-bearing mice during treatment.

Supplementary Fig. S7 Early-treatment ARK-1 xenograft stu

## Data Availability

The data generated in this study are available upon request from the corresponding author.

## References

[1] Siegel RL , Kratzer TB, Giaquinto AN, Sung H, Jemal A. Cancer statistics, 2025. CA Cancer J Clin 2025;75:10–45. 10.3322/caac.21871. PMC11745215 39817679

[2] National Cancer Institute . SEER cancer stat facts: uterine cancer (corpus Uteri). 2025. [cited 2026 Oct 9] Available from: https://seer.cancer.gov/statfacts/html/corp.html

[3] American Cancer Society . Cancer facts & figures 2025. Atlanta (GA): American Cancer Society; 2025 [cited 2025 Sept 29]. Available from: https://www.cancer.org/content/dam/cancer-org/research/cancer-facts-and-statistics/annual-cancer-facts-and-figures/2025/2025-cancer-facts-and-figures-acs.pdf.

[4] Previs RA , Coleman RL, Harris AL, Sood AK. Molecular pathways: translational and therapeutic implications of the Notch signaling pathway in cancer. Clin Cancer Res 2015;21:955–61. 10.1158/1078-0432.CCR-14-0809. PMC4333206 25388163

[5] Bray SJ , Bigas A. Modes of Notch signalling in development and disease. Nat Rev Mol Cell Biol 2025;26:522–37. 10.1038/s41580-025-00835-2. 40065190

[6] Chen C , Du Y, Nie R, Wang S, Wang H, Li P. Notch signaling in cancers: mechanism and potential therapy. Front Cell Dev Biol 2025;13:1542967. 10.3389/fcell.2025.1542967. PMC11882598 40052152

[7] Ghosh A , Mitra AK. Metastasis and cancer associated fibroblasts: taking it up a Notch. Front Cell Dev Biol 2023;11:1277076. 10.3389/fcell.2023.1277076. PMC10806909 38269089

[8] Zhou B , Lin W, Long Y, Yang Y, Zhang H, Wu K, . Notch signaling pathway: architecture, disease, and therapeutics. Signal Transduct Target Ther 2022;7:95. 10.1038/s41392-022-00934-y. PMC8948217 35332121

[9] You WK , Schuetz TJ, Lee SH. Targeting the DLL/Notch signaling pathway in cancer: challenges and advances in clinical development. Mol Cancer Ther 2023;22:3–11. 10.1158/1535-7163.MCT-22-0243. PMC9808372 36223541

[10] Zhdanovskaya N , Firrincieli M, Lazzari S, Pace E, Scribani Rossi P, Felli MP, . Targeting Notch to maximize chemotherapeutic benefits: rationale, advanced strategies, and future perspectives. Cancers (Basel) 2021;13:5106. 10.3390/cancers13205106. PMC8533696 34680255

[11] Muthuraman N , Thomas A, Samuel Ram T, Mohankumar KM, Abraham P. Is there any difference in stem cell population between type I and type II endometrial cancer? A pilot study. J Mother Child 2025;29:10–9. 10.34763/jmotherandchild.20252901.d-24-00041. PMC12153073 40433700

[12] Xiu M , Wang Y, Li B, Wang X, Xiao F, Chen S, . The role of Notch3 signaling in cancer stemness and chemoresistance: molecular mechanisms and targeting strategies. Front Mol Biosci 2021;8:694141. 10.3389/fmolb.2021.694141. PMC8237348 34195229

[13] Polychronidou G , Kotoula V, Manousou K, Kostopoulos I, Karayannopoulou G, Vrettou E, . Mismatch repair deficiency and aberrations in the Notch and Hedgehog pathways are of prognostic value in patients with endometrial cancer. PLoS One 2018;13:e0208221. 10.1371/journal.pone.0208221. PMC6283658 30521558

[14] Cho S , Lu M, He X, Ee PL, Bhat U, Schneider E, . Notch1 regulates the expression of the multidrug resistance gene ABCC1/MRP1 in cultured cancer cells. Proc Natl Acad Sci U S A 2011;108:20778–83. 10.1073/pnas.1019452108. PMC3251103 22143792

[15] Cancer Genome Atlas Research Network; Kandoth C, Schultz N, Cherniack AD, Akbani R, Liu Y, Shen H, . Integrated genomic characterization of endometrial carcinoma. Nature 2013;497:67–73. 10.1038/nature12113. PMC3704730 23636398

[16] Devor EJ , Miecznikowski J, Schickling BM, Gonzalez-Bosquet J, Lankes HA, Thaker P, . Dysregulation of miR-181c expression influences recurrence of endometrial endometrioid adenocarcinoma by modulating NOTCH2 expression: an NRG Oncology/Gynecologic Oncology Group study. Gynecol Oncol 2017;147:648–53. 10.1016/j.ygyno.2017.09.025. PMC5698180 28969912

[17] Dixit G , Gonzalez-Bosquet J, Skurski J, Devor EJ, Dickerson EB, Nothnick WB, . FGFR2 mutations promote endometrial cancer progression through dual engagement of EGFR and Notch signalling pathways. Clin Transl Med 2023;13:e1223. 10.1002/ctm2.1223. PMC10172618 37165578

[18] Byron SA , Gartside M, Powell MA, Wellens CL, Gao F, Mutch DG, . FGFR2 point mutations in 466 endometrioid endometrial tumors: relationship with MSI, KRAS, PIK3CA, CTNNB1 mutations and clinicopathological features. PLoS One 2012;7:e30801. 10.1371/journal.pone.0030801. PMC3285611 22383975

[19] Dutt A , Salvesen HB, Chen TH, Ramos AH, Onofrio RC, Hatton C, . Drug-sensitive FGFR2 mutations in endometrial carcinoma. Proc Natl Acad Sci U S A 2008;105:8713–7. 10.1073/pnas.0803379105. PMC2438391 18552176

[20] Pollock PM , Gartside MG, Dejeza LC, Powell MA, Mallon MA, Davies H, . Frequent activating FGFR2 mutations in endometrial carcinomas parallel germline mutations associated with craniosynostosis and skeletal dysplasia syndromes. Oncogene 2007;26:7158–62. 10.1038/sj.onc.1210529. PMC2871595 17525745

[21] Krakstad C , Birkeland E, Seidel D, Kusonmano K, Petersen K, Mjøs S, . High-throughput mutation profiling of primary and metastatic endometrial cancers identifies KRAS, FGFR2 and PIK3CA to be frequently mutated. PLoS One 2012;7:e52795. 10.1371/journal.pone.0052795. PMC3531332 23300780

[22] Hu W , Liu T, Ivan C, Sun Y, Huang J, Mangala LS, . Notch3 pathway alterations in ovarian cancer. Cancer Res 2014;74:3282–93. 10.1158/0008-5472.CAN-13-2066. PMC4058356 24743243

[23] McAuliffe SM , Morgan SL, Wyant GA, Tran LT, Muto KW, Chen YS, . Targeting Notch, a key pathway for ovarian cancer stem cells, sensitizes tumors to platinum therapy. Proc Natl Acad Sci U S A 2012;109:E2939–48. 10.1073/pnas.1206400109. PMC3491453 23019585

[24] Bhattacharya R , Ghosh A, Mukhopadhyay S. High-grade serous ovarian carcinoma, the “Achiles’ hill” for clinicians and molecular biologists: a molecular insight. Mol Biol Rep 2023;50:9511–9. 10.1007/s11033-023-08760-3. 37737967

[25] Park JT , Chen X, Tropè CG, Davidson B, Shih I-M, Wang T-L. Notch3 overexpression is related to the recurrence of ovarian cancer and confers resistance to carboplatin. Am J Pathol 2010;177:1087–94. 10.2353/ajpath.2010.100316. PMC2928943 20671266

[26] Chen X , Thiaville MM, Chen L, Stoeck A, Xuan J, Gao M, . Defining NOTCH3 target genes in ovarian cancer. Cancer Res 2012;72:2294–303. 10.1158/0008-5472.CAN-11-2181. PMC3342447 22396495

[27] Park JT , Li M, Nakayama K, Mao TL, Davidson B, Zhang Z, . Notch3 gene amplification in ovarian cancer. Cancer Res 2006;66:6312–8. 10.1158/0008-5472.CAN-05-3610. 16778208

[28] Allen F , Maillard I. Therapeutic targeting of Notch signaling: from cancer to inflammatory disorders. Front Cell Dev Biol 2021;9:649205. 10.3389/fcell.2021.649205. PMC8194077 34124039

[29] McCaw TR , Inga E, Chen H, Jaskula-Sztul R, Dudeja V, Bibb JA, . Gamma secretase inhibitors in cancer: a current perspective on clinical performance. Oncologist 2021;26:e608–21. 10.1002/onco.13627. PMC8018325 33284507

[30] Moore G , Annett S, McClements L, Robson T. Top Notch targeting strategies in cancer: a detailed overview of recent insights and current perspectives. Cells 2020;9:1503. 10.3390/cells9061503. PMC7349363 32575680

[31] Lehal R , Zaric J, Vigolo M, Urech C, Frismantas V, Zangger N, . Pharmacological disruption of the Notch transcription factor complex. Proc Natl Acad Sci U S A 2020;117:16292–301. 10.1073/pnas.1922606117. PMC7368267 32601208

[32] Hanna GJ , Stathis A, Lopez-Miranda E, Racca F, Quon D, Leyvraz S, . A phase I study of the pan-Notch inhibitor CB-103 for patients with advanced adenoid cystic carcinoma and other tumors. Cancer Res Commun 2023;3:1853–61. 10.1158/2767-9764.CRC-23-0333. PMC10501326 37712875

[33] Lake RJ , Haynes MK, Dreval K, Bilkis R, Sklar LA, Fan HY. A novel flow cytometric assay to identify inhibitors of RBPJ-DNA interactions. SLAS Discov 2020;25:895–905. 10.1177/2472555220932552. PMC7442708 32567455

[34] Lake RJ , Nikeghbal P, Lagutina IV, Leslie KK, Steinkamp MP, Fan HY. Auranofin synergizes with cisplatin in reducing tumor burden of NOTCH-dependent ovarian cancer. Cancer Res Commun 2025;5:1796–808. 10.1158/2767-9764.CRC-25-0190. PMC12512110 40985842

[35] Ranbhise JS , Singh MK, Ju S, Han S, Yun HR, Kim SS, . The redox paradox: cancer’s double-edged sword for malignancy and therapy. Antioxidants (Basel) 2025;14:1187. 10.3390/antiox14101187. PMC12561025 41154495

[36] Dreval K , Lake RJ, Fan HY. HDAC1 negatively regulates selective mitotic chromatin binding of the Notch effector RBPJ in a KDM5A-dependent manner. Nucleic Acids Res 2019;47:4521–38. 10.1093/nar/gkz178. PMC6511865 30916347

[37] Lake RJ , Boetefuer EL, Tsai PF, Jeong J, Choi I, Won KJ, . The sequence-specific transcription factor c-Jun targets Cockayne syndrome protein B to regulate transcription and chromatin structure. PLoS Genet 2014;10:e1004284. 10.1371/journal.pgen.1004284. PMC3990521 24743307

[38] Lake RJ , Tsai PF, Choi I, Won KJ, Fan HY. RBPJ, the major transcriptional effector of Notch signaling, remains associated with chromatin throughout mitosis, suggesting a role in mitotic bookmarking. PLoS Genet 2014;10:e1004204. 10.1371/journal.pgen.1004204. PMC3945225 24603501

[39] Abdalbari FH , Telleria CM. The gold complex auranofin: new perspectives for cancer therapy. Discov Oncol 2021;12:42. 10.1007/s12672-021-00439-0. PMC8777575 35201489

[40] Wakabayashi N , Skoko JJ, Chartoumpekis DV, Kimura S, Slocum SL, Noda K, . Notch-Nrf2 axis: regulation of Nrf2 gene expression and cytoprotection by notch signaling. Mol Cell Biol 2014;34:653–63. 10.1128/MCB.01408-13. PMC3911489 24298019

[41] Paul MK , Bisht B, Darmawan DO, Chiou R, Ha VL, Wallace WD, . Dynamic changes in intracellular ROS levels regulate airway basal stem cell homeostasis through Nrf2-dependent Notch signaling. Cell Stem Cell 2014;15:199–214. 10.1016/j.stem.2014.05.009. PMC4127166 24953182

[42] Deepika N , Rajendra Prasad N, Radhiga T. Auranofin sensitizes breast cancer cells to paclitaxel mediated cell death via regulating FOXO3/Nrf2/Keap1 signaling pathway. Cell Biochem Funct 2024;42:e3903. 10.1002/cbf.3903. 38269508

[43] Liu YP , Yang CJ, Huang MS, Yeh CT, Wu AT, Lee YC, . Cisplatin selects for multidrug-resistant CD133^+^ cells in lung adenocarcinoma by activating Notch signaling. Cancer Res 2013;73:406–16. 10.1158/0008-5472.CAN-12-1733. 23135908

[44] Dai G , Deng S, Guo W, Yu L, Yang J, Zhou S, . Notch pathway inhibition using DAPT, a gamma-secretase inhibitor (GSI), enhances the antitumor effect of cisplatin in resistant osteosarcoma. Mol Carcinog 2019;58:3–18. 10.1002/mc.22873. 29964327

[45] Koucka K , Spalenkova A, Seborova K, Tesarova T, Ehrlichova M, Krus I, . Molecular impact of NOTCH signaling dysregulation on ovarian cancer progression, chemoresistance, and taxane response. Biomed Pharmacother 2025;191:118532. 10.1016/j.biopha.2025.118532. PMC12499955 40925231

[46] Leung SOA , Konstantinopoulos PA. Advances in the treatment of platinum resistant epithelial ovarian cancer: an update on standard and experimental therapies. Expert Opin Investig Drugs 2021;30:695–707. 10.1080/13543784.2021.1939305. 34082614

