# Supplementary material for: Auranofin Potentiates Cisplatin Response in the Setting of Context-Dependent NOTCH-Associated Signaling States in Endometrial Cancer
Source: Cancer Res Commun. 2026 Oct 16;6(10):2422–33. doi: 10.1158/2767-9764.CRC-26-0404 (PMC13673326; doi:10.1158/2767-9764.CRC-26-0404)
Supplement: Supplementary file 1 [file crc-26-0404_supplementary_data_suppsm.pdf]

Supplementary Methods

Survival Analysis

To assess the robustness of survival associations, additional analyses were performed using median-based copy-number stratification and univariate Cox proportional hazards modeling with NOTCH3 copy-number treated as a continuous variable. Kaplan–Meier analyses were performed using the UCSC Xena Browser. Cox proportional hazards modeling was performed using NOTCH3 copy-number as a continuous variable. Median-based stratification analyses were used to compare outcomes between tumors with copy-number values above and below the cohort median.

RT-qPCR analysis of HES1 and HES4 expression

AN3CA and ARK-1 cells were treated with DMSO or auranofin (AuR) for 24 hours. Total RNA was extracted from cells using TRIzol reagent (Thermo Fisher Scientific, Waltham, MA). cDNA was synthesized using the High-Capacity cDNA Reverse Transcription Kit (Applied Biosystems, Cat. No. 4368813) according to the manufacturer’s instructions. HES1 and HES4 transcript levels were measured by quantitative real-time PCR and normalized to β-actin. Relative expression was analyzed using the $\Delta\Delta Ct$ method. Statistical comparisons were performed using two-tailed paired Student’s t-tests on ΔCt values from three independent biological experiments.
