# Supplementary material for: Auranofin Potentiates Cisplatin Response in the Setting of Context-Dependent NOTCH-Associated Signaling States in Endometrial Cancer
Source: Cancer Res Commun. 2026 Oct 16;6(10):2422–33. doi: 10.1158/2767-9764.CRC-26-0404 (PMC13673326; doi:10.1158/2767-9764.CRC-26-0404)
Supplement: Supplementary file 2 [file crc-26-0404_supplementary_fig.s1_suppsf1.pdf]

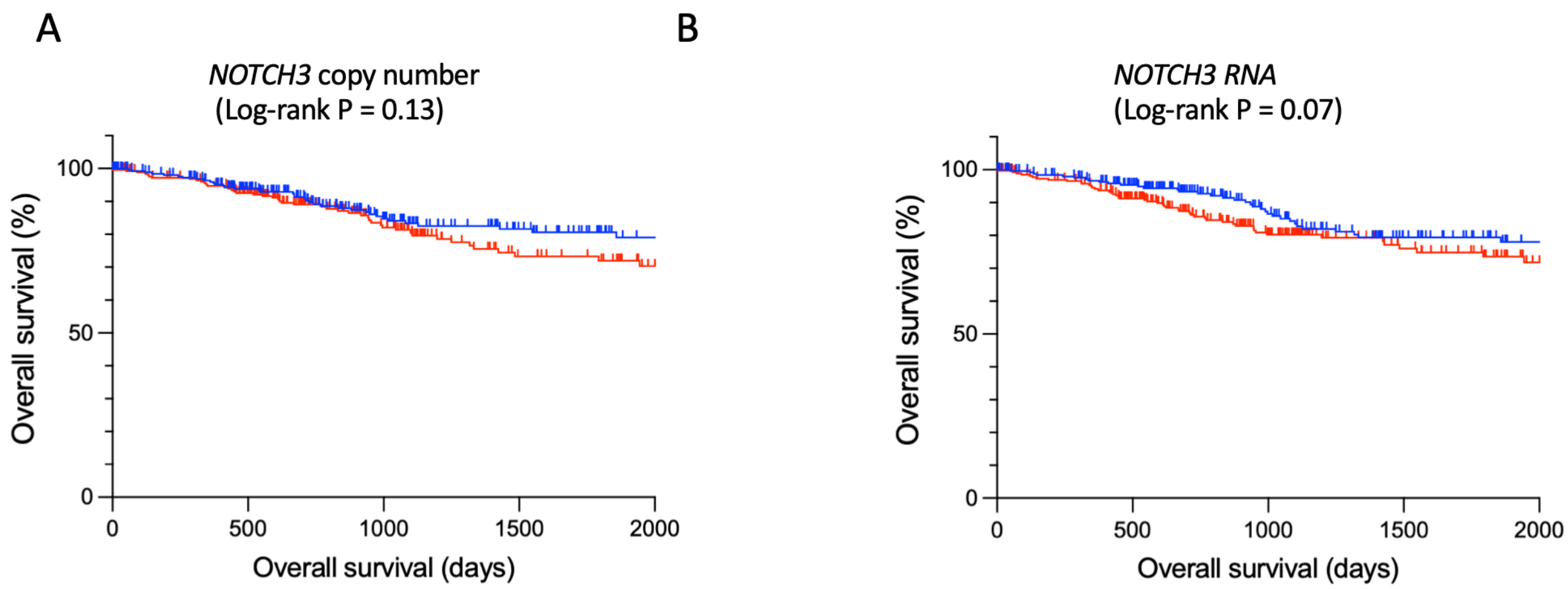

NOTCH3	0	1000	2000
Low CNV (< 50%)	269	126	50
High CNV (> 50%)	267	111	40

NOTCH3	0	1000	2000
Low RNA (< 50%)	260	121	51
High RNA (>50%)	281	119	39

Figure S1. Robustness analysis of NOTCH3 survival associations.

A, Kaplan–Meier analysis using median-based stratification of NOTCH3 copy-number demonstrates weaker survival separation than upper-versus-lower quartile grouping, indicating that the association observed in Fig. 1A is most evident among tumors with the highest levels of copy-number gain. **B,** Kaplan–Meier analysis using median-based stratification of NOTCH3 RNA expression demonstrates a similarly modest and nonsignificant association with outcome. Together with continuous Cox proportional-hazards modeling reported in the Results section, these analyses indicate that the association between NOTCH3 copy number and clinical outcome is most apparent at the upper end of the copy-number distribution and is dependent on the analytical stratification approach.
