# Supplementary material for: Auranofin Potentiates Cisplatin Response in the Setting of Context-Dependent NOTCH-Associated Signaling States in Endometrial Cancer
Source: Cancer Res Commun. 2026 Oct 16;6(10):2422–33. doi: 10.1158/2767-9764.CRC-26-0404 (PMC13673326; doi:10.1158/2767-9764.CRC-26-0404)
Supplement: Supplementary file 3 [file crc-26-0404_supplementary_fig.s2_suppsf2.pdf]

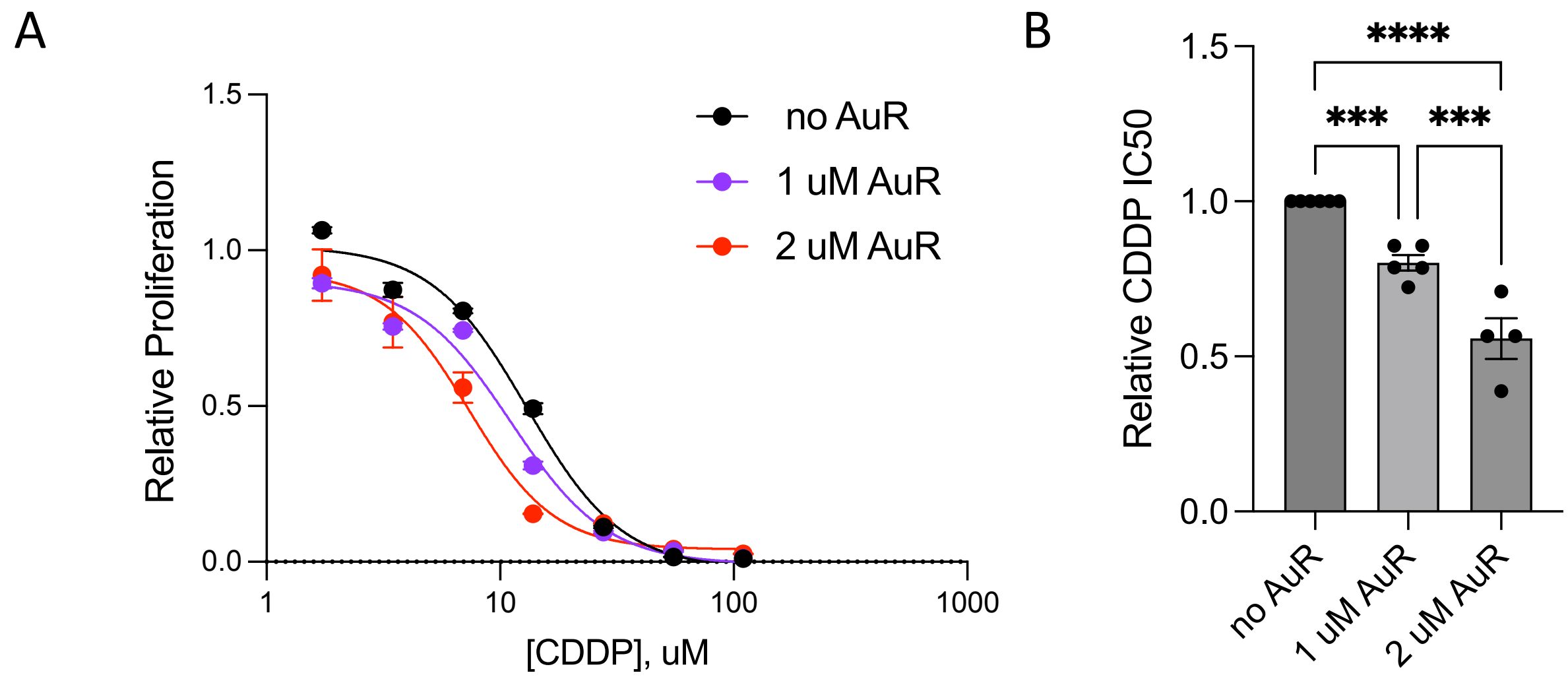

Supplementary Figure S2. AuR enhances CDDP response in additional endometrial cancer cell models.

RL95-2 cells were treated with increasing concentrations of CDDP in the absence or presence of 1 or 2 µM AuR. Relative proliferation was normalized to untreated controls. **A**, Representative nonlinear regression dose-response curves are shown at left. **B**, Quantification of relative CDDP IC50 values from independent biological replicates, with IC50 values normalized to the no-AuR condition. Data are presented as mean ± SEM from independent biological replicates. IC50 values were calculated from replicate dose-response curves. Statistical significance in panel B was determined using one-way ANOVA with multiple-comparison correction. ns, not significant; ***, *P* < 0.001; ****, P < 0.0001.
