# Supplementary material for: Auranofin Potentiates Cisplatin Response in the Setting of Context-Dependent NOTCH-Associated Signaling States in Endometrial Cancer
Source: Cancer Res Commun. 2026 Oct 16;6(10):2422–33. doi: 10.1158/2767-9764.CRC-26-0404 (PMC13673326; doi:10.1158/2767-9764.CRC-26-0404)
Supplement: Supplementary file 4 [file crc-26-0404_supplementary_fig.s3_suppsf3.pdf]

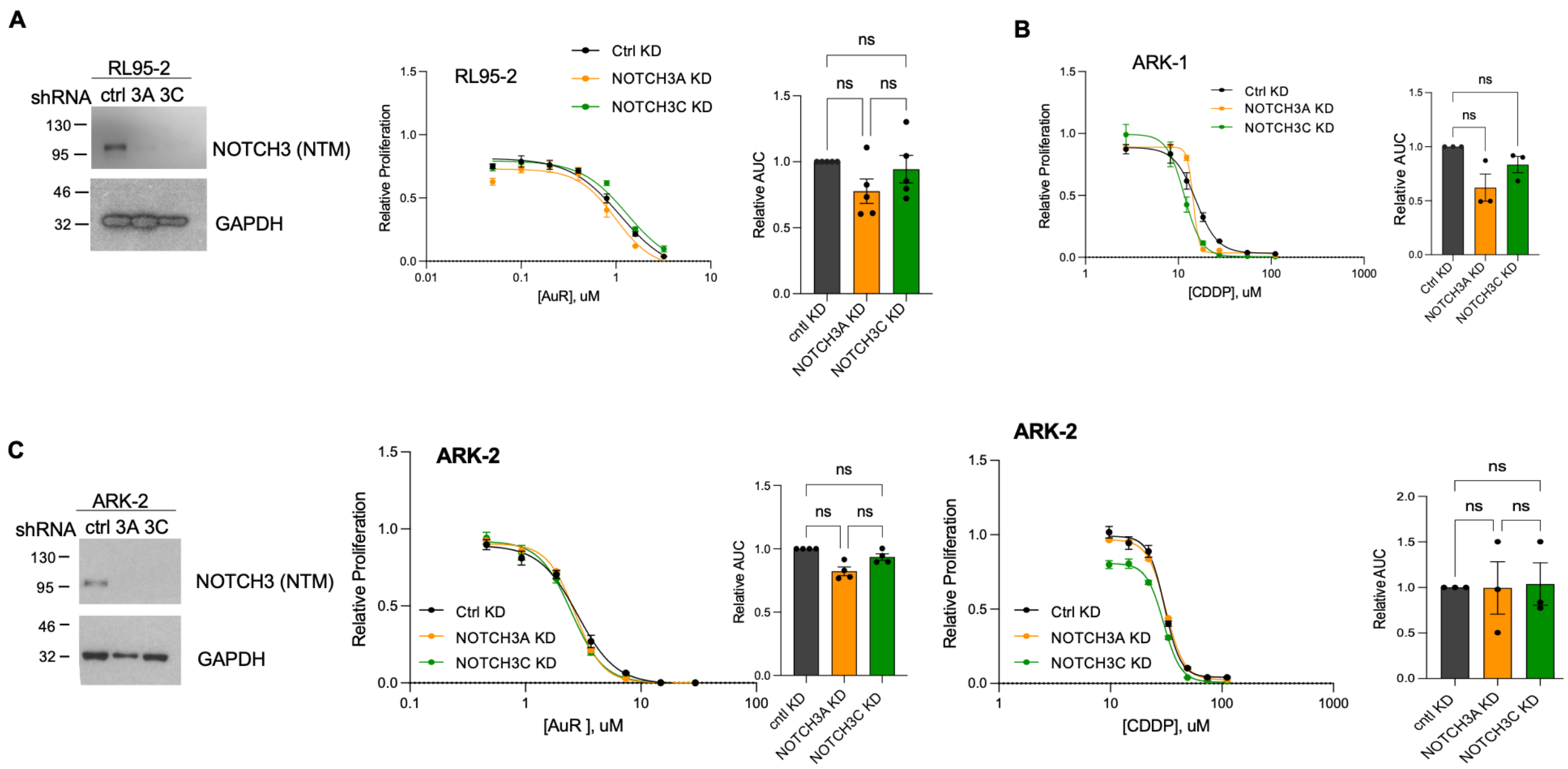

Supplementary Figure S3. NOTCH3 depletion produces minimal effects on AuR and CDDP responsiveness in additional endometrial cancer models.

A, Western blot validation of NOTCH3 knockdown and AuR dose–response analysis in RL95-2 cells. Relative proliferation curves and AUC quantification demonstrate minimal changes in AuR response following NOTCH3 depletion. **B**, CDDP dose–response analysis in ARK-1 cells following NOTCH3 knockdown. Relative proliferation curves and AUC quantification demonstrate no significant effect of NOTCH3 depletion on CDDP responsiveness. **C**, Western blot validation of NOTCH3 knockdown together with AuR and CDDP dose–response analyses in ARK-2 cells. Relative proliferation curves and corresponding AUC quantification demonstrate minimal effects of NOTCH3 depletion on either AuR or CDDP responsiveness. Data are presented as mean ± SEM from independent biological replicates. Statistical significance was determined using one-way ANOVA with multiple-comparison correction. ns, not significant.
