# Supplementary material for: Auranofin Potentiates Cisplatin Response in the Setting of Context-Dependent NOTCH-Associated Signaling States in Endometrial Cancer
Source: Cancer Res Commun. 2026 Oct 16;6(10):2422–33. doi: 10.1158/2767-9764.CRC-26-0404 (PMC13673326; doi:10.1158/2767-9764.CRC-26-0404)
Supplement: Supplementary file 5 [file crc-26-0404_supplementary_fig.s4_suppsf4.pdf]

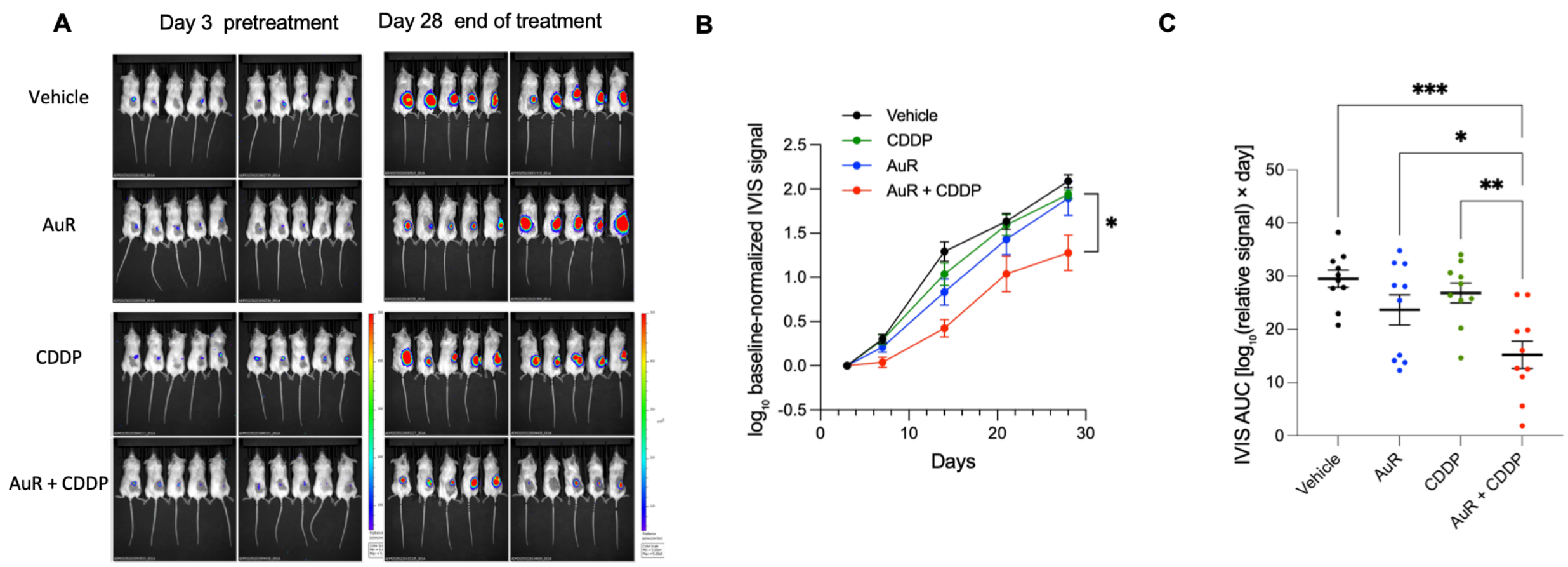

Supplementary Figure S4. Longitudinal IVIS analysis of AN3CA-Luc2 xenografts confirms enhanced tumor control with combined AuR and CDDP treatment.

A, Cohort-level IVIS images of AN3CA-Luc2 xenografts at Day 3, immediately before treatment initiation, and Day 28, at the end of treatment. Mice were treated with vehicle, auranofin (AuR), cisplatin (CDDP), or AuR + CDDP (n = 10 mice per group). **B,** Longitudinal IVIS tumor burden. IVIS signal for each mouse was normalized to its pretreatment Day 3 value and log10-transformed. Data are presented as mean ± SEM. Day 3 is shown as the pretreatment baseline; statistical analysis was performed on the post-treatment time points (Days 7, 14, 21, and 28) using two-way repeated-measures ANOVA with Geisser-Greenhouse correction. Significant effects of time ($P < 0.0001$) and treatment ($P = 0.0003$) were observed, whereas the time × treatment interaction was not significant ($P = 0.0687$). Holm-Šídák multiple-comparison testing showed significantly lower IVIS signal in the AuR + CDDP group compared with CDDP alone at Days 7, 14, and 28 (adjusted $P = 0.0249$, 0.0072, and 0.0452, respectively), but not at Day 21 ($P = 0.1517$). The Day 28 CDDP versus AuR + CDDP comparison is indicated in the graph. **C,** Cumulative IVIS tumor burden during the treatment period, quantified for each mouse as area under the curve (AUC) of the log10-transformed, baseline-normalized IVIS signal from Day 3 through Day 28. Each symbol represents one mouse; horizontal lines indicate mean ± SEM. Statistical significance was determined by one-way ANOVA with Holm-Šídák multiple-comparison correction. Adjusted P values were 0.0005 for vehicle versus AuR + CDDP, 0.0493 for AuR versus AuR + CDDP, and 0.0045 for CDDP versus AuR + CDDP. *, $P < 0.05$; **, $P < 0.01$; ***, $P < 0.001$.
