# Supplementary material for: Auranofin Potentiates Cisplatin Response in the Setting of Context-Dependent NOTCH-Associated Signaling States in Endometrial Cancer
Source: Cancer Res Commun. 2026 Oct 16;6(10):2422–33. doi: 10.1158/2767-9764.CRC-26-0404 (PMC13673326; doi:10.1158/2767-9764.CRC-26-0404)
Supplement: Supplementary file 6 [file crc-26-0404_supplementary_fig.s5_suppsf5.pdf]

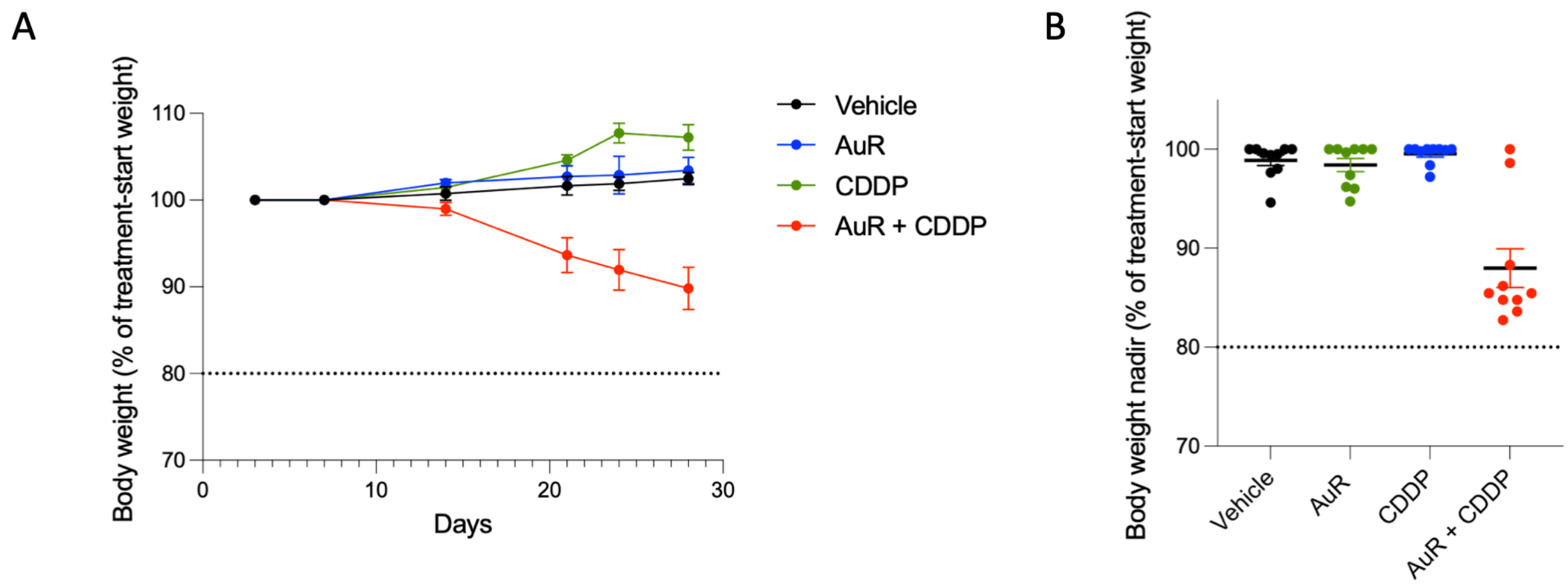

C

Treatment group	n	Mean body-weight nadir (% of treatment-start weight)	Lowest individual nadir (%)	Mice <80%
Vehicle	10	98.9 ± 0.5	94.6	0/10
AuR	10	98.4 ± 0.7	94.7	0/10
CDDP	10	99.5 ± 0.3	97.2	0/10
AuR + CDDP	10	88.0 ± 1.9	82.7	0/10

Supplementary Fig. S5. Body-weight monitoring in AN3CA xenograft-bearing mice during treatment.

A, Longitudinal body weight during the 28-day study. Body weight for each mouse was normalized to its treatment-start value and expressed as a percentage of treatment-start weight. Data are shown as mean ± SEM (n = 10 mice per group). The dotted horizontal line indicates the prespecified humane-endpoint threshold of 80% of treatment-start body weight. **B,** Body-weight nadir during the treatment period, defined as the lowest body weight recorded for each individual mouse through Day 28 and expressed as a percentage of treatment-start weight. Each point represents one mouse; bars indicate mean ± SEM. The dotted line indicates the 80% humane-endpoint threshold. **C,** Summary of body-weight nadir by treatment group, including mean ± SEM, lowest individual nadir, and the number of mice falling below 80% of treatment-start weight. Although mice receiving AuR + CDDP exhibited a greater reduction in body weight than the other treatment groups, no mouse in any group fell below the 80% humane-endpoint threshold during the treatment period.
