# Supplementary material for: Auranofin Potentiates Cisplatin Response in the Setting of Context-Dependent NOTCH-Associated Signaling States in Endometrial Cancer
Source: Cancer Res Commun. 2026 Oct 16;6(10):2422–33. doi: 10.1158/2767-9764.CRC-26-0404 (PMC13673326; doi:10.1158/2767-9764.CRC-26-0404)
Supplement: Supplementary file 8 [file crc-26-0404_supplementary_fig.s7_suppsf7.pdf]

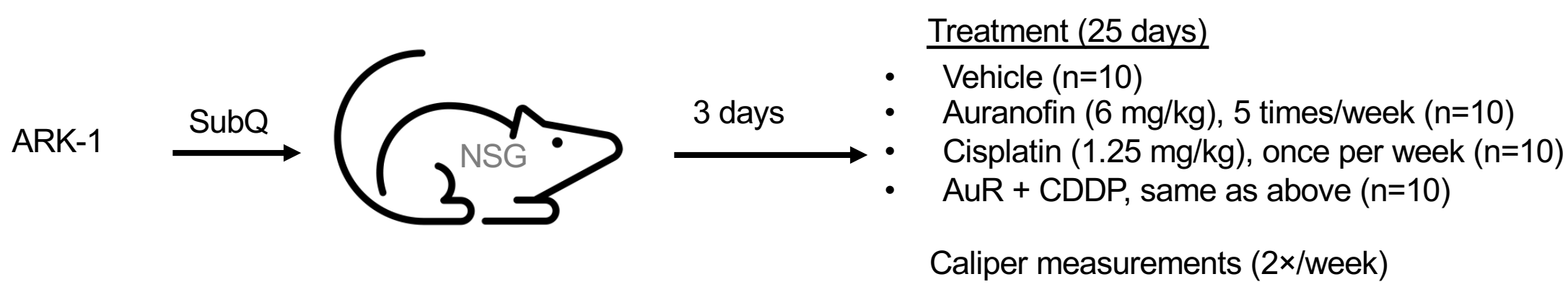

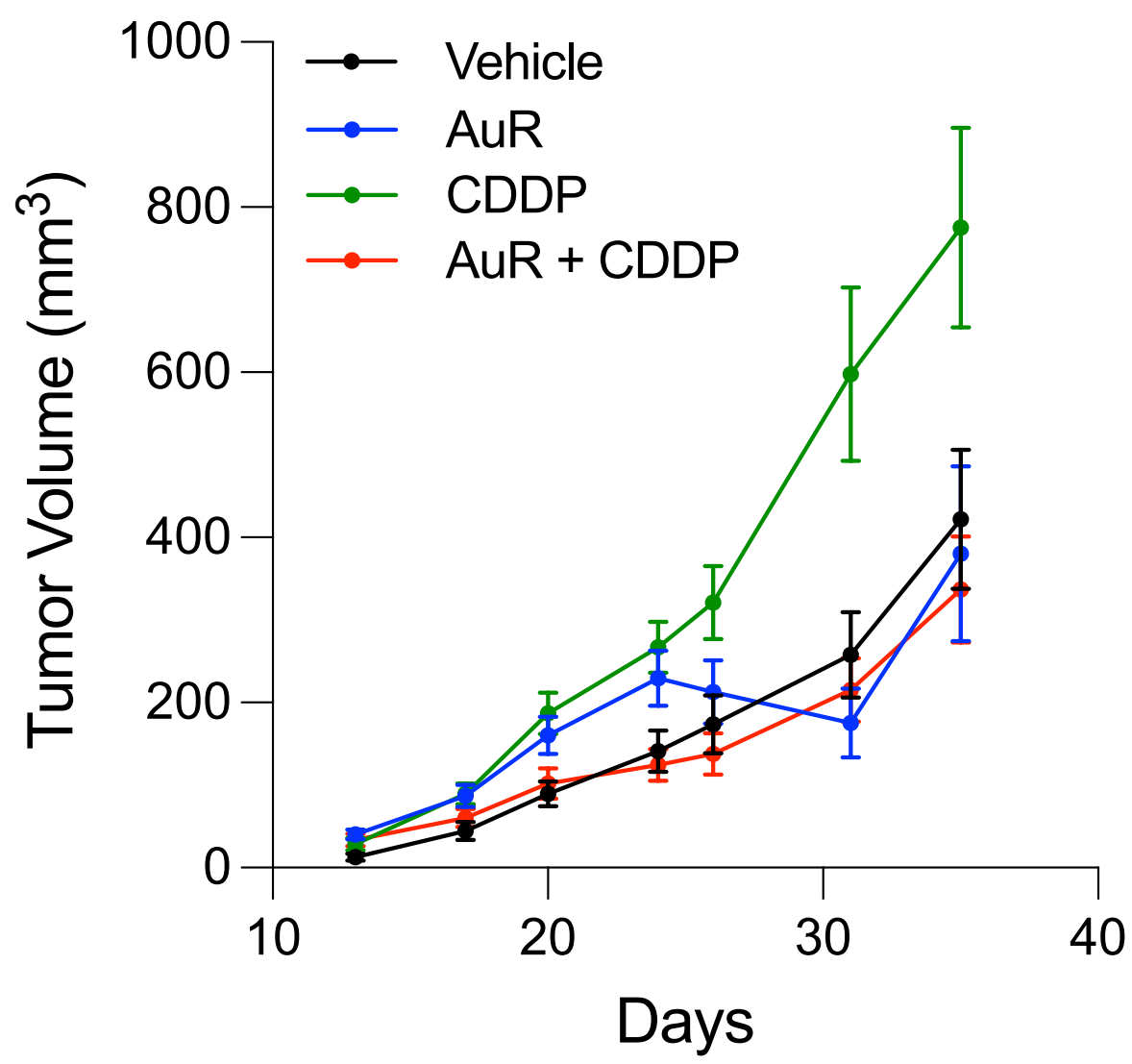

Supplementary Figure S7. Early-treatment ARK-1 xenograft study.

ARK-1 cells were implanted subcutaneously into NSG mice and treatment was initiated 3 days after implantation. Mice received vehicle, auranofin (AuR; 6 mg/kg, intraperitoneally, 5 times per week), cisplatin (CDDP; 1.25 mg/kg, intraperitoneally, once weekly), or the combination of AuR and CDDP (n = 10 mice per group). Tumor volumes were measured twice weekly by caliper. Tumor growth curves are shown as mean ± SEM tumor volume.
