# Supplementary material for: Auranofin Potentiates Cisplatin Response in the Setting of Context-Dependent NOTCH-Associated Signaling States in Endometrial Cancer
Source: Cancer Res Commun. 2026 Oct 16;6(10):2422–33. doi: 10.1158/2767-9764.CRC-26-0404 (PMC13673326; doi:10.1158/2767-9764.CRC-26-0404)
Supplement: Supplementary file 9 [file crc-26-0404_supplementary_table_s1_suppst1.pdf]

Supplementary Table S1.

Supplementary Table S1. Primers used in qPCR	
hHES1_ChIP_F	5′-CCT CCC ATT GGC TGA AAG T-3′
hHES1_ChIP_R	5′-CGG ATC CTG TGT GAT CCC TA-3′
hHES4_ChIP_F	5′-CTC AGG CCG TTT CCC TAT TT-3′
hHES4_ChIP_R	5′-CGA GGC GTG ACT GAC AGC-3′
hHES1_RT_F	5′-TGA AAG TCT GAG CCA GCT GA-3′
hHES1_RT_R	5′-GTC ACC TCG TTC ATG CAC TC- 3′
hACTb_RT_F	5′-TCG TGC GTG ACA TTA AGG AG-3′
hACTb_RT_R	5′-ATG CCA GGG TAC ATG GTG GT-3′
hHES4_RT_F	5′-CAA AAC CCT CAT CCT GGA CG-3′
hHES4_RT_R	5′-TTC ACC TCC GCC AGA CAC- 3′
