# Supplementary material for: Auranofin Potentiates Cisplatin Response in the Setting of Context-Dependent NOTCH-Associated Signaling States in Endometrial Cancer
Source: Cancer Res Commun. 2026 Oct 16;6(10):2422–33. doi: 10.1158/2767-9764.CRC-26-0404 (PMC13673326; doi:10.1158/2767-9764.CRC-26-0404)
Supplement: Supplementary file 10 [file crc-26-0404_supplementary_table_s2_suppst2.pdf]

Supplementary Table S2. Histologic and molecular characteristics of endometrial cancer cell-line models

Cell line	Reported histology / origin	Key molecular features	Molecular context / nearest TCGA-like class
AN3CA	Undifferentiated endometrial carcinoma / poorly differentiated endometrial adenocarcinoma	MSI-high/MMRd; TP53 R213Q; FGFR2 N550K; PTEN-mutant; ARID1A/ARID1B alterations	MMRd-like
RL95-2	Grade 2 moderately differentiated adenosquamous endometrial carcinoma	MSI-low; POLE-WT; copy-number-high; PTEN alterations; TP53 V218del; HRAS Q61H	Copy-number-high / p53-abnormal-like, with mixed molecular features
ARK-1	Uterine serous carcinoma	TP53 R248W; HER2/ERBB2-high	Copy-number-high / p53-abnormal-like
ARK-2	Uterine serous carcinoma	TP53 Q165*; HER2/ERBB2-high	Copy-number-high / p53-abnormal-like
KLE	Poorly differentiated endometrial carcinoma	TP53 R175H	p53-abnormal-like

Footnote: Molecular-context assignments are approximate and based on reported histologic and genomic features; they do not represent formal TCGA molecular classification of the cell lines.

Sources: AN3CA, DepMap/CCLE; Byron et al., 2008; Li et al., 2025. RL95-2, Way et al., 1983; Devor et al., 2020; DepMap/CCLE. ARK-1 and ARK-2, Yang et al., 2025. KLE, DepMap/CCLE and published TP53 characterization.

1. Li ES, Ho R, Tao R, Choi YWY, Shin CY, Chen SY*, et al.* Molecular subtyping of endometrial carcinoma cell lines uncovers subtype-specific targetable vulnerabilities. NPJ Precis Oncol **2025**;9:254
2. Byron SA, Gartside MG, Wellens CL, Mallon MA, Keenan JB, Powell MA*, et al.* Inhibition of activated fibroblast growth factor receptor 2 in endometrial cancer cells induces cell death despite PTEN abrogation. Cancer Res **2008**;68:6902-7
3. Devor EJ, Gonzalez-Bosquet J, Thiel KW, Leslie KK. Genomic characterization of five commonly used endometrial cancer cell lines. Int J Oncol **2020**;57:1348-57
4. Way DL, Grosso DS, Davis JR, Surwit EA, Christian CD. Characterization of a new human endometrial carcinoma (RL95-2) established in tissue culture. In Vitro **1983**;19:147-58
5. Yang Z, Mogre S, Jun H, He R, Ghosh Chaudhary S, Bhattarai UR*, et al.* Functional Profiling of p53 and RB Cell Cycle Regulatory Proficiency Suggests Mechanism-Driven Molecular Stratification in Endometrial Carcinoma. Cancer Res Commun **2025**;5:719-42
