# Supplementary material for: Auranofin Potentiates Cisplatin Response in the Setting of Context-Dependent NOTCH-Associated Signaling States in Endometrial Cancer
Source: Cancer Res Commun. 2026 Oct 16;6(10):2422–33. doi: 10.1158/2767-9764.CRC-26-0404 (PMC13673326; doi:10.1158/2767-9764.CRC-26-0404)
Supplement: Supplementary file 11 [file crc-26-0404_supplementary_table_s3_suppst3.pdf]

Supplementary Table S3. RT-qPCR analysis of HES1 and HES4 expression following auranofin treatment in AN3CA and ARK-1 cells.

Cell line	AuR (µM)	Gene	n	ΔCt DMSO, mean ± SD	ΔCt AuR, mean ± SD	ΔΔCt	Fold change (2^-ΔΔCt)	P value
AN3CA	1.25	HES1	3	8.544 ± 0.189	8.407 ± 0.203	−0.137	1.10	0.599
AN3CA	1.25	HES4	3	18.290 ± 0.587	18.179 ±0.343	−0.111	1.08	0.846
AN3CA	2.5	HES1	3	8.544 ± 0.189	8.116 ± 0.779	−0.428	1.35	0.476
AN3CA	2.5	HES4	3	18.290 ± 0.587	18.129 ±0.176	−0.161	1.12	0.720
ARK-1	1.25	HES1	3	6.747 ± 0.188	6.606 ± 0.424	−0.141	1.10	0.423
ARK-1	1.25	HES4	3	8.960 ± 0.380	8.869 ± 0.405	−0.091	1.07	0.423
ARK-1	2.5	HES1	3	6.747 ± 0.188	6.715 ± 0.251	−0.032	1.02	0.510
ARK-1	2.5	HES4	3	8.960 ± 0.380	9.560 ± 0.399	+0.600	0.66	0.128

[#] AN3CA and ARK-1 cells were treated with DMSO, 1.25 or 2.5 µM auranofin for 24 hours. HES1 and HES4 expression was normalized to β-actin. Fold change was calculated using the 2^-ΔΔCt method. P values were determined by two-tailed paired t-tests performed on ΔCt values from three independent experiments.
